# Myeloid-specific interferon regulatory factor 5 promotes bone formation *via* orchestration of osteoclast lineage-osteoblast coupling

**DOI:** 10.1016/j.jbc.2026.113279

**Published:** 2026-06-22

**Authors:** Huan Zhao, Xiaoyue Sun, Songqin Zhou, Zhengrui Chang, Jingwen Yang, Jingjing Yu, Zijun Wang, Yi Tang, Changdong Lin, Li Wang, Stephen J. Weiss, Lingxin Zhu

**Affiliations:** 1 State Key Laboratory of Oral & Maxillofacial Reconstruction and Regeneration, Key Laboratory of Oral Biomedicine Ministry of Education, Hubei Key Laboratory of Stomatology, School & Hospital of Stomatology, Wuhan University, Wuhan, China; 2 Division of Genetic Medicine, Department of Internal Medicine, University of Michigan, Ann Arbor, Michigan, USA; 3 Life Sciences Institute, University of Michigan, Ann Arbor, Michigan, USA; 4 Cardiopulmonary and Critical Care Rehabilitation Center, Shanghai YangZhi Rehabilitation Hospital (Shanghai Sunshine Rehabilitation Center), School of Life Sciences and Technology, Tongji University, Shanghai, China

**Keywords:** bone coupling, bone remodeling, cell crosstalk, IRF5, osteoclast

## Abstract

Bone remodeling is orchestrated by the balanced activity of bone-resorbing osteoclasts and bone-forming osteoblasts. Interferon regulatory factor 5 (IRF5), a member of the IRF family of transcription factors, serves as a critical regulator of macrophage immune-related activity. However, macrophages also serve as the precursor cell of osteoclasts where the role of IRF5 in osteoclast-mediated bone remodeling *in vivo* remains undefined. Here, we find that IRF5 exhibits nuclear localization specifically in preosteoclasts during macrophage-osteoclast transition *in vitro*. In turn, myeloid cell-specific *Irf5* conditional KO (*Irf5*^*ΔM/ΔM*^) mice exhibit a significant osteopenic phenotype. Unexpectedly, osteoclast activity remained unaltered, while osteoblastic bone formation was significantly reduced. Interestingly, while conditioned media from WT preosteoclasts and osteoclasts increased the osteogenic potential of osteoblastic cells, this stimulatory activity was largely abrogated in conditioned media recovered from *Irf5*^*ΔM/ΔM*^ preosteoclasts and osteoclasts. Further, the osteogenic potential of bone marrow stromal cells was markedly inhibited when co-cultured with *Irf5*^*ΔM/ΔM*^ osteoclasts. Complementing these findings, genome wide analysis revealed that the *Irf5-*deficient osteoclast lineage displayed major changes in transcriptional programs related to extracellular matrix organization, bone development, and Notch signaling. Taken together, our study identifies IRF5 as a previously unrecognized transcriptional regulator of osteoblast-stimulating factors within the osteoclast lineage, thereby offering new insights on osteoimmune regulation of bone remodeling.

Bone is constantly remodeled through the coupled regulation of osteoclastic bone resorption and osteoblastic bone formation. In the healthy adult skeleton, the amount of resorbed bone is generally equal to the amount of new bone formation, thus meeting structural and metabolic demands (1). Osteoclasts, the principal bone-resorbing cells, are multinucleated giant cells formed by the fusion of monocyte/macrophage in a process termed osteoclastogenesis (2). Once formed, mature osteoclasts attach tightly to bone and degrade the underlying matrix by secreting hydrochloric acid and a suite of proteolytic enzymes (3, 4, 5, 6). By contrast, osteoblasts arise from mesenchymal stem cells in the bone marrow stroma where they are responsible for bone matrix synthesis and its subsequent mineralization (5). Of note, the proper coupling between osteoclast and osteoblast lineages is required to support physiological bone remodeling (1). This bone remodeling process is carefully regulated by various local and systemic factors whose alteration in metabolic bone diseases, such as osteoporosis, arthritis, periodontitis, bone metastasis or osteopetrosis, leads to abnormalities in bone remodeling (1, 5). As such, defining the regulatory factors that control bone remodeling has important therapeutic implications for the prevention and treatment of bone disease.

Bone resorbing osteoclasts as well as immune regulatory macrophages derive from a common myeloid cell origin, share a number of molecular regulators, and have reciprocal interactions in both heath and disease (7). Interferon regulatory factors (IRFs) are a family of nine transcriptional regulators (*i.e.*, IRF1-9) that control the expression of interferon type I and interferon type I -inducible genes, playing crucial roles in modulating innate and adaptive immune responses (8). In turn, dysregulation of IRF signaling is involved in pathogenesis of many autoimmune diseases (8). Recently, the role of several IRF members, including IRF1, IRF8 and IRF9, has been linked to bone remodeling (9, 10, 11). Among IRF family members, IRF5 is a key transcription factor in defining the inflammatory macrophage phenotype, both in terms of controlling cytokine expression and regulating immune responses (12, 13, 14). However, whether IRF5 is involved in myeloid-lineage osteoclast activity and bone remodeling *in vivo* remains unclear.

Interestingly, whereas IRF5 and IRF8 act on overlapping gene sets in macrophages (15, 16), IRF8 function has also been reported to extend to bone resorption where *Irf8*^*−/−*^ mice exhibit severe osteoporosis due to enhanced osteoclast formation and activity (11). As such, we considered the possibility that IRF5 might similarly regulate osteoclast function. Here, we find that *Irf5* expression displays a dynamic profile similar with that of *Irf8* during macrophage-osteoclast transition. However, while myeloid cell-specific *Irf5* conditional KO (*Irf5*^*ΔM/ΔM*^) mice display a significant osteopenic phenotype, IRF5 proved to be dispensable for osteoclast differentiation and bone resorptive function. Instead, myeloid *Irf5* deficiency inhibited bone formation by disrupting osteoclast lineage-osteoblast coupling, thereby providing new insights into the cellular and molecular mechanisms of IRF-dependent bone remodeling.

## Results

### IRF5 displays dynamic expression profile during osteoclast differentiation

**Figure 1:**
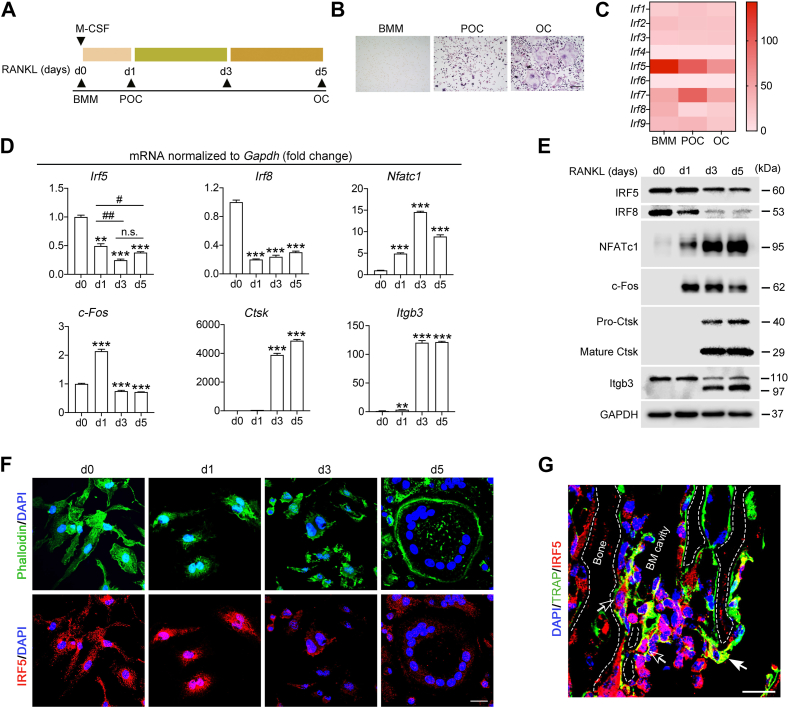
**Dynamic expression of IRF5 during osteoclastogenesis.***A*, schematic of experimental staging of osteoclast differentiation. *B*, TRAP staining of BMM, POC and OC. The scale bar represents 200 μm, n = 3. *C*, heatmap of *Irf* family expression in BMM, POC and OC by RNA-Seq analysis. *D*, relative mRNA expression of *Irf5*, *Irf8*, *Nfatc1*, *c-Fos*, *Ctsk*, and *Itgb3* in BMMs at 0, 1, 3, and 5 days after M-CSF and RANKL addition (RT-PCR). Data are presented as mean ± SD (n = 3). Data were analyzed using one-way ANOVA with Bonferroni correction. *E*, Western blot of IRF5, IRF8, NFATc1, c-Fos, Ctsk, and Itgb3 expression in BMMs at 0, 1, 3, and 5 days after M-CSF and RANKL addition (n = 3). *F*, IRF5 (*red*), phalloidin (*green*), and DAPI (*blue*) staining performed over the course of BMMs differentiation into OCs. The scale bar represents 20 μm, n = 3. *G*, IRF5 (*red*) and TRAP (*green*) immunofluorescence of a femur section from 3–4-week-old WT male mice. The *white solid arrow points* to osteoclasts on the bone surface, while the *white hollow arrow* indicates preosteoclasts on the bone surface. The scale bar represents 20 μm, n = 3. ∗∗*p* < 0.01 *versus* d0; ∗∗∗*p* < 0.001 *versus* d0; #*p* < 0.05 *versus* d1; ##*p* < 0.01 *versus* d1; n.s., no significance. BMM, bone marrow-derived macrophage; POC, preosteoclast; IRF, interferon regulatory factor; TRAP^+^, tartrate-resistant acid phosphatase; OC, osteoclast

Following stimulation with M-CSF and RANKL, bone marrow-derived macrophages (BMMs) first commit to tartrate-resistant acid phosphatase-positive (TRAP^+^) mononuclear cells (preosteoclasts) that subsequently fuse to form TRAP^+^ multinuclear osteoclasts (17). To assess the expression profile of *Irf* family members during this progression (Fig. 1, *A* and *B*), RNA sequencing demonstrated that *Irf5* levels were highest in BMMs relative to other *Irf* family members and subsequently decreased in preosteoclasts and osteoclasts (Fig. 1*C*). Likewise, in agreement with earlier studies (11), *Irf8* levels similarly decreased during osteoclast commitment (Fig. 1, *D* and *E*). Dynamic changes in *Irf5* expression occur in inverse fashion with the increased expression of other well-known osteoclast maturation makers, including NFATc1, c-Fos, Ctsk and Itgb3 (18, 19), during osteoclastogenesis at both mRNA and protein levels (Figs. 1, *D* and *E* and S1*A*).

While IRF5 protein levels were highest in BMMs, its nuclear localization was confined to the preosteoclast stage of differentiation with IRF5 excluded from the nucleus of mature osteoclasts (Fig. 1*F*). Extending these data into the *in vivo* setting, only limited IRF5 expression could be localized to the cytoplasm of osteoclasts adherent to the bone surface, while high nuclear IRF5 localization was confined to TRAP^+^ mononuclear preosteoclasts (Fig. 1*G*). By contrast, IRF5 was not detected in osteoblasts either *in vitro* or *in vivo* (Fig. S2, *A*–*C*). Taken together, IRF5 exhibits dynamic changes in its expression and intracellular localization during osteoclast differentiation.

### Myeloid *Irf5* conditional KO mice exhibit a decreased bone mass phenotype

**Figure 2:**
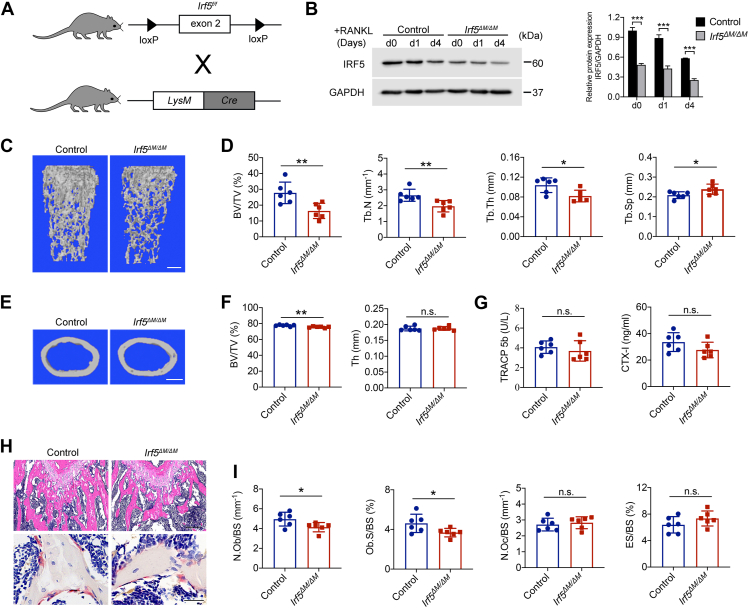
**Conditional KO of myeloid *Irf5* decreases bone mass in male mice.***A*, pattern diagram of *Irf5*^*ΔM/ΔM*^ transgenic mouse generation. *B*, IRF5 expression as assessed by Western blot at indicated time during BMM differentiation into osteoclasts (n = 3). *C*, representative three-dimensional (3D) reconstruction of distal femur trabeculae of 3-month-old control and *Irf5*^*ΔM/ΔM*^ male mice. Scale bars, 500 μm, n = 6. *D*, quantification of trabecular bone volume, trabecular number, trabecular bone thickness, trabecular separation of trabeculae as determined by μCT in 3-month-old male control and *Irf5*^*ΔM/ΔM*^ mice (n = 6). *E*, representative three-dimensional (3D) reconstruction of cortical bones of 3-month-old control and *Irf5*^*ΔM/ΔM*^ male mice are shown. The scale bar represents 500 μm, n = 6. *F*, quantification of trabecular bone volume and Th of cortical bones as determined by μCT in 3-month-old male control and *Irf5*^*ΔM/ΔM*^ mice (n = 6). *G*, serum TRACP 5b and CTX-I levels in 3-month-old male control and *Irf5*^*ΔM/ΔM*^ mice (n = 6). *H*, H&E (*up*) and TRAP (*down*) staining of distal femur trabeculae of 3-month-old male control and *Irf5*^*ΔM/ΔM*^ mice. The scale bar represents 200 μm (H&E), 40 μm (TRAP), n = 6. *I*, quantification of osteoblast number per bone surface, osteoblast surface per bone surface, osteoclast number per bone surface and eroded surface per bone surface as determined by H&E and TRAP staining (n = 6). Data are presented as mean ± SD. ∗*p* < 0.05, ∗∗*p* < 0.01, ∗∗∗*p* < 0.001, n.s., no significance. Data were analyzed using two-way ANOVA with Bonferroni correction (*B*) and unpaired Student’s *t* test (*D*, *F*–*I*).

Having identified changes in IRF5 expression during the macrophage-osteoclast transition, we generated a myeloid-specific *Irf5* conditional KO mouse model by crossing *Irf5*^*flox/flox*^ with *LysM*-Cre mice (*i.e.*, *LysM-Cre/Irf5*^*flox/flox*^ and hereafter referred to as *Irf5*^*ΔM/Δ*M^) wherein Cre is expressed in myeloid progenitors that include the osteoclast-derived lineage (20) (Fig. 2*A*). We then assessed bone morphometry of 3-month-old *Irf5*^*ΔM/ΔM*^ mice and control littermates. Though IRF5 levels were decreased by approximately 50% (Fig. 2*B*), *Irf5*^*ΔM/ΔM*^ male mice exhibited an obvious osteoporosis-like phenotype in distal femurs (Fig. 2, *C*–*I*). Further, μCT scans of the distal femurs of male mice demonstrated a markedly decreased femoral trabecular bone volume (BV/TV), trabecular number, and trabecular bone thickness, but with increased trabecular separation relative to control male mice (Fig. 2, *C* and *D*). The cortical bone volume was reduced slightly, but significantly, in *Irf5*^*ΔM/ΔM*^ male mice relative to control mice, while cortical thickness was unaltered (Fig. 2, *E* and *F*). *Irf5*^*ΔM/ΔM*^ female mice, however, showed no overall bone mass phenotype (Fig. S3, *A*–*D*), indicating that myeloid *Irf5* positively regulates bone mass in a gender-specific fashion.

### IRF5 is dispensable for osteoclast differentiation and bone-resorptive function *in vitro* and *in vivo*

**Figure 3:**
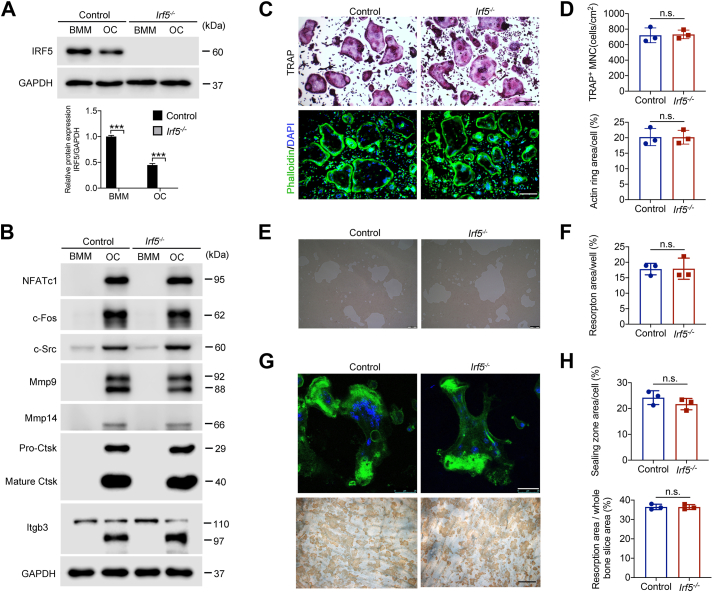
**IRF5-independent regulation of *in vitro* osteoclastogenesis and resorptive function.***A*, IRF5 expression in BMMs and osteoclasts from control and *Irf5*^*−/−*^ mice as assessed by Western blot (n = 3). *B*, expression of NFATc1, c-Fos, c-Src, Mmp9, Mmp14, Ctsk, and Itgb3 in BMMs and OCs of control and *Irf5*^*−/−*^ mice (n = 3). *C*, representative images of TRAP staining (*up*) and FITC-conjugated phalloidin (*green*) staining (*down*) of control and *Irf5*^*−/−*^ OCs (n = 3). DAPI (*blue*). The scale bar represents 200 μm. *D*, quantification of TRAP-positive multinucleated cells (*up*) and actin ring area (*down*) (n = 3). *E*, bone-resorption pits generated by control and *Irf5*^*−/−*^ OCs (n = 3). The scale bar represents 100 μm. *F*, quantification of resorption pits area (n = 3). *G*, control and *Irf5*^*−/−*^ OCs were cultured atop bone slices for 6 days followed by FITC-conjugated phalloidin (*green*) and DAPI (*blue*) staining (n = 3). The scale bar represents 25 μm (*up*). Following osteoclast removal, resorption pits were visualized by WGA-3,3′-diaminobenzidine staining (n = 3). The scale bar represents 200 μm (*down*). *H*, quantification of sealing zone and resorption pit area (n = 3). Data are presented as mean ± SD; n.s., no significance. Data were analyzed using two-way ANOVA with Bonferroni correction (*A*) and unpaired Student’s *t* test (*D, F*, and *H*). OC, osteoclast

Given the osteopenic state of *Irf5*-targeted mice, we first elected to characterize the osteoclastogenic status of these cells *in vitro* (Fig. S4, *A*–*E*). *Irf5*^*ΔM/ΔM*^ BMMs undergo osteoclastogenesis comparably to control cells across a range of RANKL concentrations and initial cell densities as assessed by TRAP staining (Fig. S4, *A*–*D*). Considering the residual expression of *Irf5* in *Irf5*^*ΔM/ΔM*^ BMMs (Fig. 2*B*), we next constructed an *Irf5* total KO (*Irf5*^*−/−*^) mouse model and confirmed the complete loss of expression in *Irf5*^*−/−*^ BMMs (Fig. 3*A*). Upon osteoclastogenic induction, neither the number of TRAP^+^ multinucleated cells nor F-actin ring area was altered in the *Irf5*^*−/−*^ group (Fig. 3, *C* and *D*). Likewise, both gene and protein expression levels of osteoclast differentiation- and maturation-related markers, including *NFATc1, c-Fos, c-Src, Mmp9, Mmp14, Ctsk* and *Itgb3* (18, 19, 21), were indistinguishable between *Irf5*^*−/−*^ and WT cells (Figs. 3*B* and S5, *A* and *B*).

To next assess the bone resorptive activity of *Irf5*^*−/−*^ osteoclasts, we cultured WT and *Irf5*^*−/−*^ BMMs with M-CSF and RANKL for 6 days on bone matrices in order to monitor the formation of resorption pits. Under these conditions, the matrix-resorbing function of wild-type *versus Irf5*^*−/−*^ osteoclasts appeared identical (Fig. 3, *E* and *F*). In addition to demineralization, osteoclasts must proteolyze collagen fibrils deposited in the bone matrix (22). As such, we further induced WT and *Irf5*^*−/−*^ BMMs into osteoclasts atop bovine cortical bone slices. When osteoclasts attach to bone surface, they polarize and undergo significant morphological changes, including the formation of an F-actin ring that defines the bone resorption microenvironment (*i.e.*, the "sealing zone") where bone is actively resorbed (6, 23). Here, neither the sealing zone area nor the size of bone resorption lacunae were altered in *Irf5*^*−/−*^ osteoclasts (Fig. 3, *G* and *H*).

Given that osteoblast-lineage cells produce RANKL (encoded by *Tnfsf11*) and OPG (encoded by *Tnfrsf11b*) to regulate osteoclastogenesis, and the RANKL/OPG ratio represents the most established mechanism controlling osteoclast development and activity (24), we assessed the expression of RANKL and OPG *in vivo*. In this regard, immunofluorescence analyses showed that RANKL and OPG expression on bone surfaces were comparable between *Irf5*^*ΔM/ΔM*^ male mice and control mice, with no significant differences observed (Fig. S6, *A* and *B*). Accordingly, the RANKL/OPG ratio remained unchanged between the two groups (Fig. S6*B*). Similarly, *in vitro*, osteoblasts derived from bone marrow stromal cells (BMSCs) recovered from *Irf5*^*ΔM/ΔM*^ mice and controls expressed comparable levels of *Tnfsf11* and *Tnfrsf11b*, leaving the *Tnfsf11*/*Tnfrsf11b* ratio unchanged (Fig. S6*C*). These results indicate that the preserved RANKL/OPG ratio maintains normal regulatory feedback on osteoclastogenesis, which is consistent with the unaltered osteoclast function observed in *Irf5*-deficient mice. Complementing these results, despite the decreased bone mass observed in *Irf5*^*ΔM/ΔM*^ male mice, osteoclast numbers and the surface area of bone erosions were comparable to control mice (Fig. 2, *H* and *I*), markers of osteoclast-mediated bone resorption, serum TRAcP5b, and the type I collagen degradation product, CTX-1 (25), were unaltered in *Irf5*^*ΔM/ΔM*^ male mice relative to controls (Fig. 2*G*). As expected, the levels of osteoclast differentiation and bone resorption function in *Irf5*^*ΔM/ΔM*^ female mice were unaffected when compared to control female mice (Fig. S3, *E*–*G*). Taken together, these data indicate that myeloid IRF5 does not exert significant effects on osteoclastic bone resorption.

### Impaired bone formation contributes to the osteopenic phenotype in *Irf5*^*ΔM/ΔM*^ mice

**Figure 4:**
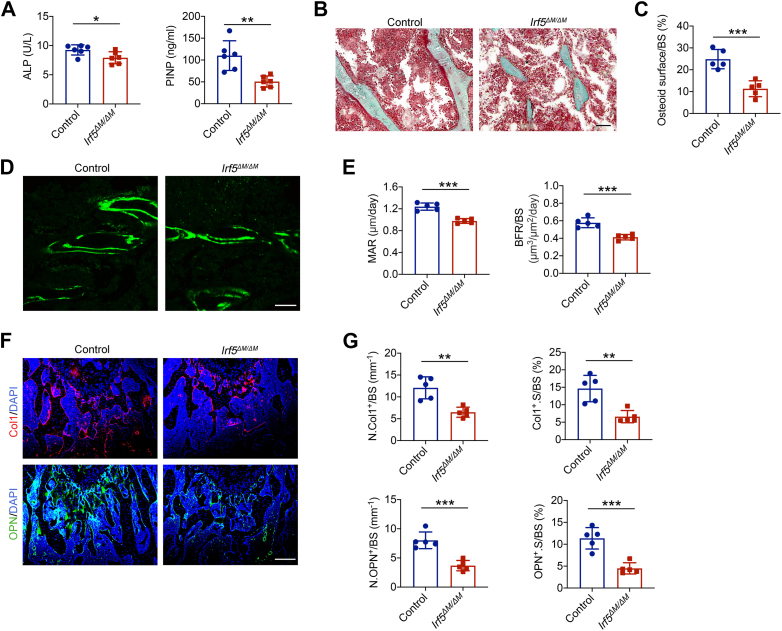
**Conditional KO of myeloid *Irf5* inhibits bone formation *in vivo*.***A*, serum alkaline phosphatase and procollagen type 1 N-terminal propeptide levels in 3-month-old male control and *Irf5*^*ΔM/ΔM*^ mice (n = 6). *B*, representative images of Golden’s trichrome staining of the distal femurs of 3-month-old male control and *Irf5*^*ΔM/ΔM*^ mice. The scale bar represents 50 μm, (n = 5). *C*, quantification of osteoid surface/BS of the distal femur trabeculae of 3-month-old male control and *Irf5*^*ΔM/ΔM*^ mice (n = 5). *D*, double calcein bone labeling and representative confocal images of distal femur trabeculae of 3-month-old male control and *Irf5*^*ΔM/ΔM*^ mice (n = 5). The Scale bar represents 50 μm. *E*, quantification of mineral apposition rate and BFR/BS as assessed in 3-month-old male control and *Irf5*^*ΔM/ΔM*^ mice (n = 5). *F*, representative images of immunofluorescence staining of Col1 (*red*) or OPN (*green*) with DAPI (*blue*) of the distal femur trabeculae of 3-month-old male control and *Irf5*^*ΔM/ΔM*^ mice (n = 5). The scale bar represents 100 μm. *G*, quantification of N.Col1^+^/BS, Col1^+^.S/BS, N.OPN^+^/BS, OPN^+^.S/BS of the distal femur trabeculae of 3-month-old male control and *Irf5*^*ΔM/ΔM*^ mice (n = 5). Data are presented as mean ± SD. ∗*p* < 0.05, ∗∗*p* < 0.01, ∗∗∗*p* < 0.001, n.s., no significance. All data were analyzed using unpaired Student’s *t* test.

As bone homeostasis is maintained *in vivo* by the functional coupling of osteoclastic bone resorption and osteoblastic bone formation (26), we next monitored osteoblastic bone formation activity in *Irf5*^*ΔM/ΔM*^ mice. Serum levels of alkaline phosphatase and procollagen type 1 N-terminal propeptide (PINP), reflecting bone-forming activity *in vivo* (27, 28), were significantly decreased in *Irf5*^*ΔM/ΔM*^ male mice (Fig. 4*A*). Consistent with these serological results, the bone formation rate and mineral apposition rate were likewise decreased in *Irf5*^*ΔM/ΔM*^ male mice as assessed by double calcein bone labeling (Fig. 4, *D* and *E*). Higher-magnification images of Goldner’s trichrome staining demonstrate a 55% reduction in osteoid formation on trabecular surfaces in *Irf5*^*ΔM/ΔM*^ male mice (Fig. 4, *B* and *C*). Strikingly, consistent with decreased osteoblast number and osteoblast surface per bone surface in *Irf5*^*ΔM/ΔM*^ mice showed by H&E staining (Fig. 2, *H* and *I*), Col1^+^ and OPN^+^ osteoblasts were significantly reduced in *Irf5*^*ΔM/ΔM*^ male mice (Fig. 4, *F* and *G*). Hence, dampened bone formation is responsible for the osteopenic phenotype detected in *Irf5*^*ΔM/ΔM*^ mice.

As BMSCs represent a mesenchymal progenitor population that both supports hematopoiesis and gives rise to osteoblasts (29), we sought to determine whether the availability of osteoblast progenitors contributes to the bone phenotype observed in *Irf5*^*ΔM/ΔM*^ mice. As such we performed colony-forming unit assays for fibroblasts and osteoblasts to assess the osteogenic potential of BMSCs (30). Here, we found that the formation of colony-forming unit assays for fibroblasts and osteoblasts were comparable between *Irf5*^*ΔM/ΔM*^ mice and the control group (Fig. S7, *A*–*D*). Further, no differences were observed in the number of SP7^+^ osteoblast progenitors (31) found on bone surfaces between *Irf5*^*ΔM/ΔM*^ mice and control mice (Fig. S7, *E* and *F*). Taken together, myeloid-specific *Irf5* deficiency does not impair the generation or abundance of osteoblast progenitors, suggesting that the reduced osteoblastic bone formation observed in *Irf5*^*ΔM/ΔM*^ mice is not attributable to deficits in BMSC-derived osteoblast precursor populations.

Independent of these findings, given that IRF5 controls the expression of multiple inflammatory cytokines (*e.g.*, *Il1b*, *Il6*, *Il12b* and *Tnf*) and can shape innate immune responses (13, 32, 33), we considered the possibility that *Irf5* targeting triggered an inflammatory response that might have influenced the bone phenotype observed in our model. In this regard, under basal conditions, the expression levels of *Il1b*, *Il6*, *Il12b* and *Tnf* were uniformly low and comparable between *Irf5*^*ΔM/ΔM*^ and control cells *in vitro* (Fig. S8*C*). Upon LPS stimulation, while control BMMs exhibited a robust induction of these inflammatory cytokines, this response was markedly impaired in *Irf5*^*ΔM/ΔM*^ cells (Fig. S8*C*). Further, the limited distribution of inflammatory F4/80^+^CD11c^+^ macrophages in bone marrow (13) was unaffected in *Irf5*^*ΔM/ΔM*^ mice relative to controls (Fig. S8, *A* and *B*). As such, *Irf5* deletion did not appear to trigger an inflammatory phenotype under physiological conditions.

### Osteoclast lineage IRF5 expression promotes osteoblastic cell migration and mineralization

**Figure 5:**
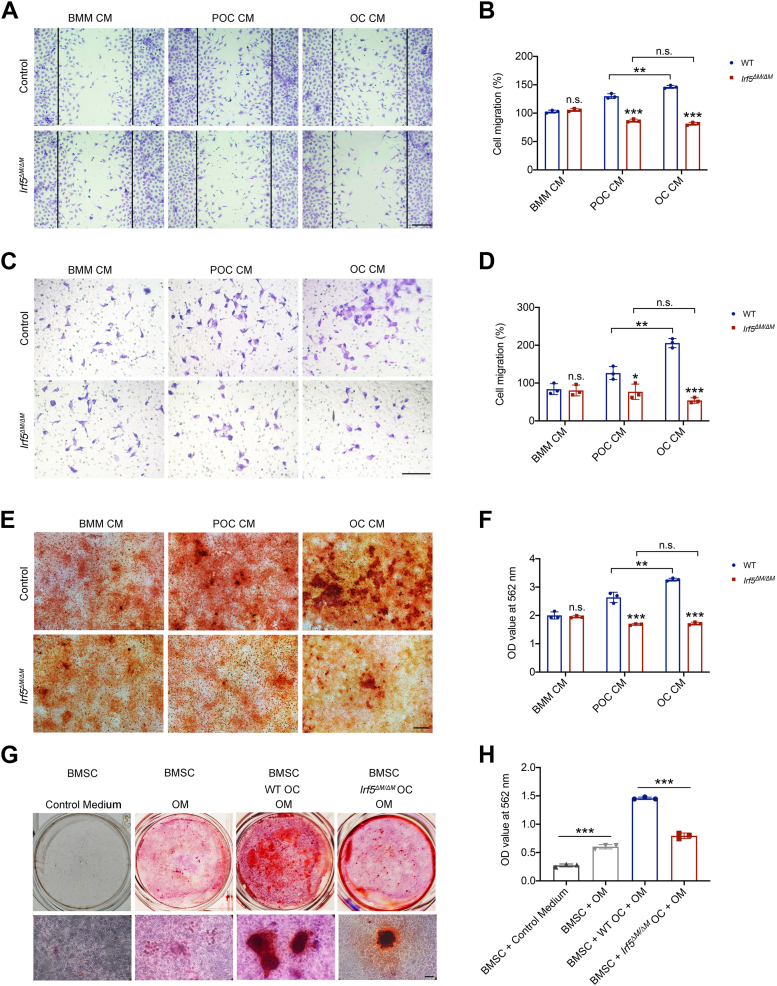
***Irf5*^*ΔM/ΔM*^ osteoclasts fail to support the migratory and mineralization activities of osteoblasts *in vitro*.***A*, 2D migration of Mc3T3-E1 cells following culture with serum-free CM recovered from BMM, POC or OC cells generated by control and *Irf5*^*ΔM/ΔM*^ mice was evaluated as shown following crystal *violet* staining (n = 3). The scale bar represents 200 μm. *B*, the number of migrated cells is presented as percentages relative to the control group (n = 3). *C*, vertical migration of Mc3T3-E1 cells following culture with serum-free CM recovered from BMM, POC or OC cells generated from control and *Irf5*^*ΔM/ΔM*^ mice in a Transwell migration assay with *crystal violet* staining (n = 3). The scale bar represents 200 μm. *D*, the number of migrated cells is presented as percentages relative to the control group (n = 3). *E*, representative images of Alizarin red staining for Mc3T3-E1 cells cultured with CM of BMM, POC or OC cells generated from control and *Irf5*^*ΔM/ΔM*^ mice in the presence of osteogenic medium in a 1:1 ratio for 21 days (n = 3). The scale bar represents 1 mm. *F*, quantitative analysis of the bound alizarin red of Mc3T3-E1 cells by measuring the optical density at 562 nm (n = 3). *G*, representative images of Alizarin red staining for bone marrow stromal cell-derived osteoblasts cocultured with OC cells from control or *Irf5*^*ΔM/ΔM*^ mice in the presence of osteogenic medium for 21 days (n = 3). *H*, quantitative analysis of Alizarin red deposits generated by bone marrow stromal cell-derived osteoblasts as determined by optical density at 562 nm (n = 3). ∗*p* < 0.05, ∗∗*p* < 0.01, ∗∗∗*p* < 0.001, n.s., no significance. Data were analyzed using two-way ANOVA with Bonferroni correction *(B, D*, and *F*) or one-way ANOVA with Bonferroni correction (*H*) and presented as mean ± SD. conditioned medium; POC, preosteoclast; OC, osteoclast

Given the high expression and specific nuclear localization of IRF5 in preosteoclasts (Fig. 1, *E* and *F*), we speculated that IRF5 potentially regulates bone mass by controlling crosstalk between osteoclasts and osteoblasts. To test the hypothesis, we performed *in vitro* studies to investigate the coupling phenomenon between these cell populations. While conditioned medium recovered from either control preosteoclasts or osteoclasts remarkably increased motility and Transwell migration of osteoblastic MC3T3-E1 cells, this stimulatory activity was lost in conditioned medium generated by *Irf5*^*ΔM/ΔM*^ preosteoclasts or osteoclasts (Fig. 5, *A*–*D*). Further, relative to control conditioned medium, *Irf5*^*ΔM/ΔM*^ preosteoclasts and osteoclasts conditioned medium only weakly stimulated the mineralizing activity of MC3T3-E1 cells as assessed by alizarin red staining (Fig. 5, *E* and *F*). The stimulatory activity generated by control osteoclasts was significantly stronger than that of preosteoclasts, while the activity of conditioned medium recovered from the *Irf5*^*ΔM/ΔM*^ osteoclasts was decreased and more similar to that of the *Irf5*^*ΔM/ΔM*^ preosteoclasts (Fig. 5, *A*–*F*). These findings were corroborated in direct coculture experiments of BMSCs from WT mice with osteoclasts from *Irf5*^*ΔM/ΔM*^ or control mice, respectively. Following 21 days in co-culture in the presence of mineralizing induction medium, Alizarin red staining indicated that the osteogenic potential of BMSCs was inhibited significantly when cocultured with *Irf5*^*ΔM/ΔM*^ osteoclasts relative to control osteoclasts (Fig. 5, *G* and *H*), indicating IRF5 regulates the direct crosstalk between osteoclasts and osteoblasts.

**Figure 6:**
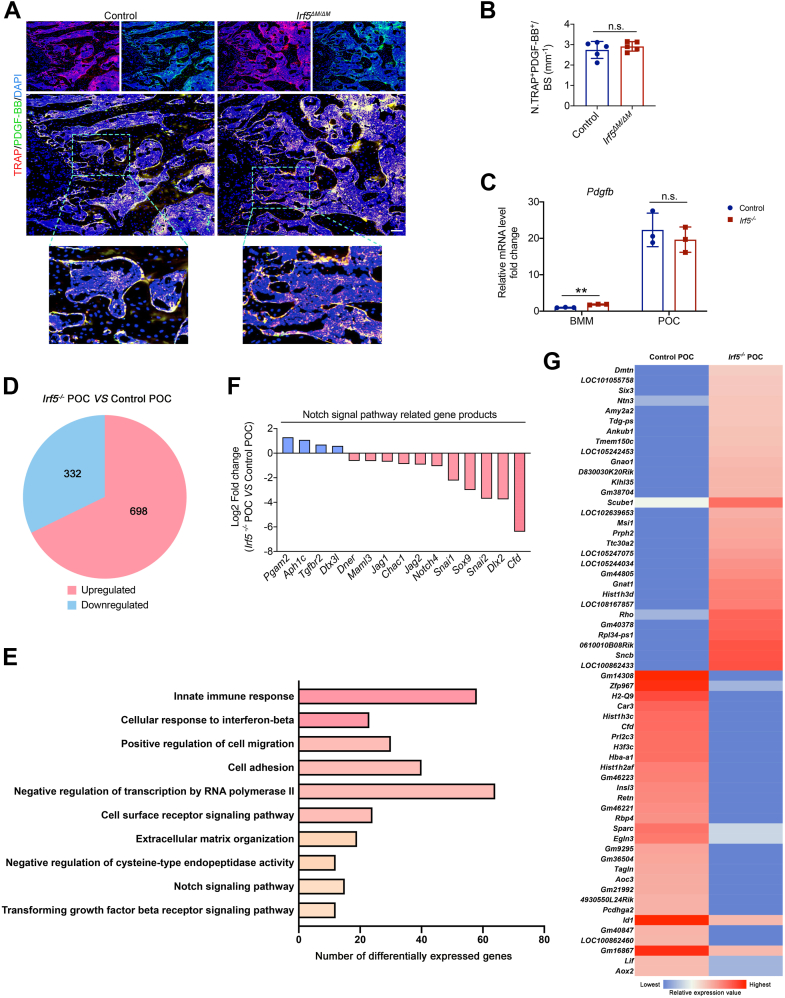
**PDGF-BB expression and genome wide analysis of transcriptional programs activated in *Irf5*^*−/−*^ preosteoclasts.***A*, Representative images of immunofluorescent staining of TRAP (*red*) and PDGF-BB (*green*) with DAPI staining (*blue*) of the distal femur trabeculae of 3-month-old male control and *Irf5*^*ΔM/ΔM*^ mice (n = 5). ThScale bar, 100 μm. *B*, quantification of N.TRAP^+^PDGF-BB^+^/BS of the distal femur trabeculae of 3-month-old male control and *Irf5*^*ΔM/ΔM*^ mice (n = 5), data were analyzed using unpaired Student’s *t* test. *C*, relative mRNA expressions of *Pdgfb* in BMM and POC cells of control and *Irf5*^*−/−*^ group (n = 3). Data were analyzed using two-way ANOVA with Bonferroni correction and presented as mean ± SD. n.s., no significance. *D*, pie chart illustrates the distribution of total transcripts altered in *Irf5*^*−/−*^ POC relative to control POC cells. *E*, DAVID Gene Ontology analysis of differentially expressed genes from *Irf5*^*−/−*^ POC *versus* control POC cells. *F*, expression of differentially expressed genes between *Irf5*^*−/−*^ POC and control POC cells enriched in Notch signal pathway-linked genes by DAVID Gene Ontology analysis. *G*, the 30 most highly upregulated and downregulated transcripts in *Irf5*^*−/−*^ POC cells as compared with control POC cells are shown. POC, preosteoclast.

Independent of direct osteoclast-osteoblast crosstalk, preosteoclasts has been reported to stimulate H-type blood vessel formation in bone by releasing PDGF-BB, thus facilitating new bone formation (17, 34, 35, 36). However, while IRF5 can bind to the *Pdgfb* promoter in other cell types (37), *Pdgfb* expression in *Irf5-*deficient preosteoclasts was comparable to control preosteoclasts (Fig. 6*C*), and the number of TRAP^+^/PDGF-BB ^+^ cells in trabecular bone was not altered in *Irf5*^*ΔM/ΔM*^ mice (Fig. 6, *A* and *B*). Hence, at this juncture, we favor a mechanism wherein IRF5-competent pre-/osteoclasts directly impact osteoblast function.

### *Irf5-*dependent control of preosteoclast/osteoclast transcriptional programs

To assess the global impact of *Irf5* targeting developing osteoclasts, gene expression was determined in WT *versus Irf5* KO preosteoclasts and osteoclasts. In preosteoclasts, *Irf5* targeting altered the expression of 1030 unique transcripts with a minimum of 1.5-fold change, including 698 up regulated genes and 332 down regulated genes (Fig. 6*D*), the 30 most highly upregulated and downregulated genes in *Irf5* KO preosteoclasts as compared with control preosteoclasts are presented (Fig. 6*G*). By contrast, 400 differentially expressed genes were found in *Irf5-*deficient osteoclasts relative to control osteoclasts (Fig. S9, *A*–*C*). Using DAVID Gene Ontology analysis, several important biological processes were enriched by differentially expressed genes between *Irf5*^*−/−*^ and control preosteoclasts (Fig. 6*E*), such as innate immune response, cellular response to interferon-β, negative regulation of transcription by RNA polymerase II, cell surface receptor signaling pathways, and the Notch signaling pathway. Interestingly, we noted changes in the expression of a series of Notch signaling pathway-related transcripts, including *Notch4*, *Jag1*, *Jag2*, *Maml3*, *Dner, Sox9,* and *Snai1* (38, 39) (Fig. 6*F*) that has been reported to be involved in the coupling between angiogenesis and osteogenesis (17, 34, 35, 40). Meanwhile, categories of extracellular matrix organization, inflammatory response, and bone development are enriched in comparisons between *Irf5*^*−/−*^ and control osteoclasts (Fig. S9*B*). Consequently, while these data underline the complex role played by IRF5 in regulating the coupling between osteoclast lineage cells and osteoblasts, further studies will be required to identify the dominant effectors controlling bone formation *in vivo*.

## Discussion

Bone is not only a crucial component of the skeletal-locomotor system, but also functions as an immunological organ that harbors hematopoietic stem and immune progenitor cells (41). Studies on bone phenotypes of immunocompromised mice have helped demonstrate the inseparable link existing between the immune and skeletal systems (7). Members of the IRF family have been found to play nonnegligible roles in regulating osteoclastogenesis and bone metabolism (9, 10, 11). While IRF5 functions as a crucial regulator of macrophage phenotypes (13, 14), potential roles for IRF5 in osteoclast differentiation and function have not been previously explored. It has long been recognized that there are extensive coupling interactions between osteoclasts and osteoblasts during bone remodeling that serve to preserve bone balance (1). Here, we unexpectedly find myeloid *Irf5* supports bone homeostasis by controlling osteoclast lineage-osteoblast coupling *in vivo* while leaving osteoclast differentiation and bone resorptive functions intact.

As an autoimmune susceptibility gene, IRF5 is well recognized as a central regulator of inflammatory macrophage phenotypes, controlling the expression of numerous cytokines and shaping innate immune responses (42). IRF5 is typically localized in the cytoplasmic compartment, and is activated by phosphorylation or ubiquitination, which results in enhanced nuclear translocation and gene regulation (42, 43). In this study, though IRF5 specifically localized to the nucleus at the preosteoclast phase, the transcription factor was dispensable for osteoclast differentiation and bone-resorbing function both *in vitro* and *in vivo*. These findings contrast with recent study describing effects of IRF5 targeting on osteoclast formation *in vitro* (44), but this report relied on siRNA delivery in primary macrophage cells where potential off-target effects may explain the apparent discrepancies.

During bone remodeling, new bone formation frequently occurs in areas where resorption has previously taken place (45). At these sites, temporary anatomical structures are assembled, termed basic multicellular units, that include osteoclasts, osteoblasts, osteocytes, bone lining cells and capillary blood vessels (46). Within these structures, osteocytes and bone lining cells both originate from mature osteoblasts (45, 46, 47). A preceding resorption phase is a prerequisite for the initiation of subsequent bone formation, with the processes balanced by osteoclast-osteoblast coupling that ensure the precise formation of new bone at sites of resorption to meet structural and metabolic demands during this process (25). Osteoclast lineage cells of at different phases of differentiation are able to signal bone formation by cell–cell contact or secretory factors. For example, osteoclasts can regulate the migration and differentiation of osteoblasts by secreting soluble factors such as S1P, CTHRC1 and C3 (45). In turn, preosteoclasts coupling bone formation by secreting PDGF-BB and inducing H-type blood vessel formation (17, 36, 48, 49). However, with regard to the latter point, *pdgfb* expression was unaffected in *Irf5*^*−/−*^ preosteoclasts as well as TRAP^+^/PDGF-BB ^+^ cells in *Irf5*^*ΔM/ΔM*^ mice.

The complexity of the transcriptional changes detected in KO preosteoclasts and osteoclasts preclude the simple identification of the key stimulatory – or inhibitory effectors operating *in vitro* or *in vivo*. Furthermore, it is unlikely that the detected changes in the preosteoclast and/or osteoclast transcriptomes do not exert effects outside osteoblastogenic programs alone. Although *Pdgfb* expression was unchanged in *Irf5-*deficient preosteoclasts, we detected a series of differentially expressed genes enriched in the Notch signaling pathway, which can also be involved in vessel growth in postnatal long bone as well as osteogenesis coupling (50). In addition, osteal macrophages reside within bone lining tissues *in vivo* where they are located immediately adjacent to osteoblasts, forming a distinctive canopy structure that covers mature osteoblasts (46, 51, 52). As osteal macrophages are able to regulate bone formation and express myeloid lineage markers (53), further work is needed to determine whether IRF5 targeting affects their function. That having been said, our osteoblast-osteoclast co-culture system supports our contention that direct interactions can take place between these two populations independent of a potential accessory role for osteomacs. Further, while conditioned media recovered from IRF5-expressing pre-/osteoclasts clearly impacts osteoblast function, the relative roles of soluble *versus* extracellular vesicle-derived effectors, such as those arising from exosomes, microvesicles, and apoptotic bodies, remains to be determined (48, 54, 55).

Other IRF family members have been reported to exert inhibitory effects on osteoclast formation, and mice deficient in *Irf1*, *Irf8* and *Irf9* each display reduced bone density and increased osteoclastogenesis (9, 10, 11). The dynamic expression profile of IRF5 during macrophage-osteoclast transition *in vitro* and bone mass phenotype of *Irf5*^*ΔM/ΔM*^ mice *in vivo* are similar to those observed in *Irf8*-targeted mice, but their regulatory effects on osteoclasts are strikingly different. IRF8 suppresses osteoclastogenesis by inhibiting the function and expression of NFATc1 (11). Similarly, IRF8 and IRF5 rapidly translocate to the nucleus following CpG stimulation in human plasmacytoid dendritic cells and act on overlapping gene sets, while exerting opposing functions on TLR9 signaling (16). In microglial cells, IRF8 positively regulates IRF5 expression and activity (56). By contrast, we find that myeloid IRF5 and IRF8 differentially regulate osteoclast formation and activity. As such, the regulation and functional roles assumed by these two transcription factors are cell-type-specific. IRF1 and IRF8 have been reported to cooperate with IRF5 in regulating macrophage polarization (15). Interestingly, *Irf1*^*−/−*^ bone marrow macrophages exhibit enhanced osteoclast activity and bone resorptive function *in vitro* (10), but potential roles in regulating osteoclast-osteoblast crosstalk remain to be determined.

In summary, the current study identifies the positive role of myeloid IRF5 in regulating physiological bone mass in mice and provides new insights into the cellular and molecular mechanisms involved in skeletal homeostasis. Given the fundamental importance of immune regulators in bone health and disease (7), the identification of IRF5 as the crucial regulator of osteoclast-mediated bone remodeling could prove useful in helping to develop therapeutic strategies targeting metabolic bone diseases. Indeed, while IRF5 inhibitors have been developed to ameliorate various inflammatory and autoimmune disease states (42, 57), the possibility that IRF5 targeting could exert deleterious on bone metabolism requires careful consideration.

## Experimental procedures

### Mouse strains

Transgenic mice were purchased from Jackson Laboratory: *Irf5*^*flox/flox*^ (*Irf5*^*f/f*^, stock number: #017311), *LysM*-Cre (stock number: #004781) and *E2a*-Cre (stock number: #003314). C57BL/6J WT mice were purchased from the Experimental Animal Center of Hubei Province. Myeloid cell-specific *Irf5* KO mice (*LysM*-Cre/*Irf5*^*f/f*^, hereafter referred to as *Irf5*^*ΔM/ΔM*^) were created by crossing the *Irf5*^*f/f*^ mice with the *LysM*-Cre transgenic mice (58). To obtain *Irf5* total KO mice (hereafter referred to as *Irf5*^−/−^), *E2a*-Cre mice were bred with *Irf5*^*f/f*^ mice to generate *E2a*-Cre/*Irf5*^*+/−*^, then crossing *E2a*-Cre/*Irf5*^*+/−*^ mice with WT mice to generate *Irf5*^*+/−*^ mice, *Irf5*^*+/−*^ mice were then crossed to generate *Irf5*^*−/−*^ mice and littermate controls (*Irf5*^*+/+*^). All mice were on a pure C57BL/6 background. All animal studies were approved by the Institutional Animal Care and Use Committee at School & Hospital of Stomatology, Wuhan University.

### Radiographic analysis

Excised 3-month-old mouse femurs were fixed with 4% paraformaldehyde for 24 h. Three-dimensional Micro-CT was performed using a SkyScan 1176 (Bruker, Kontich) with a resolution ratio of 9 μm. The skeletal parameters assessed by micro-CT followed published nomenclature guidelines (59). Trabecular bone microarchitecture, including the BV/TV, trabecular bone thickness, trabecular number, and trabecular separation, were analyzed in a manually delineated region of interest 0.36 mm–3.6 mm proximal to the distal femoral growth plate. Cortical parameters, including BV/TV and cortical bone thickness, the 0.9 mm region of the femoral mid-diaphysis were analyzed.

### Bone histomorphometry analysis

After scanning for μ-CT, samples were decalcified in 10% EDTA for 4 weeks, embedded with paraffin, and 4 μm coronal serial sections were stained with H&E and TRAP (Sigma-Aldrich, 387A-1 KT). Mineral apposition rate and bone formation rate were determined with double-calcein bone labeling as described (6). Briefly, 3-month old mice were given calcein (20 mg/kg, Sigma, C0875) in 2% NaHCO_3_ intraperitoneally 9 and 2 days before dissection, plastic sections (10 μm) were made, observed under confocal laser scanning microscope (Olympus), and followed by Golden trichrome staining. All parameters of bone histomorphometry were measured on the basis of the Report of the American Society of Bone and Mineral Research Histomorphometry Nomenclature Committee (60).

### Tissue immunofluorescence

For the deparaffinized sections staining, sections were rehydrated, covered overnight with one or a combination of two primary antibodies that included Col1 (Abcam, ab270993, 1:500), OPN (Santa Cruz, sc-21742, 1:500), PDGF-BB (Santa Cruz, sc-365805, 1:500), TRAP (Santa Cruz, sc-30833, 1:500), RANKL (Santa Cruz, sc-9073, 1:500), OPG (Santa Cruz, sc-8408, 1:500), F4/80 (Abcam, ab300421, 1:500), CD11c (Ebioscience, 14,011,481, 1:500), and SP7 (Santa Cruz, sc-393325,1:500) followed by incubating with species-specific secondary antibodies directed against the primary antibodies, including Dylight 594-conjugated goat anti-rabbit immunoglobulin G (Abbkine, A23420), Dylight 488-conjugated donkey anti-mouse immunoglobulin G (Abbkine, A24211), Alexa Fluor 647 Donkey anti-Goat IgG (Antgene, ANT033S), Alexa Fluor 488 Donkey anti-Goat IgG (Antgene, ANT025) at 37 °C for 1 h, then the sections were washed and mounted with a DAPI-containing mounting medium (ZSGB-BIO), negative controls were performed by replacing primary antibodies with nonimmune bovine serum (61).

For the frozen sections staining, femurs from 4-weeks old male mice were fixed in 4% paraformaldehyde at 4 °C overnight, decalcified in 0.5 M EDTA and transferred to 20% sucrose which included 2% PVP for 24 h, then embedded in OCT (Sakura), and 40 μm frozen sections were made, permeabilized with 0.3% Triton X-100 in PBS for 15 min, blocked with 2.5% BSA for 1 h at 37 °C. Samples were then treated with primary antibodies directed against TRAP (Santa Cruz, sc-517428, 1:500) or SP7 (Santa Cruz, sc-393325,1:500) with IRF5 (Abcam, ab181553, 1:1000) overnight at 4 °C. The covered sections were washed and incubated with dylight 594-conjugated goat anti-rabbit immunoglobulin G and Dylight 488-conjugated donkey anti-mouse immunoglobulin G at 37 °C for 1 h. After further washes with PBS, the sections were mounted with a DAPI-containing mounting solution.

### Serum measurements

Mice were deprived of food and water for 6 h before sacrifice, and the serum levels of TRAcP, C-terminal telopeptide of type I collagen, alkaline phosphatase and PINP were quantified using TRAcP 5b ELISA kit (Immunodiagnostic Systems, SB-TR103), RapLaps™ (C-terminal telopeptide of type I collagen) EIA (Immunodiagnostic Systems, AC-06F1), Alkaline Phosphatase Assay Kit (Colorimetric) (abcam, ab83369) and PINP EIA kit (Immunodiagnostic Systems, AC-33F1), respectively, according to the manufacturer’s instructions.

### Osteoclasts differentiation assay and F-actin ring analysis

BMMs and mature osteoclasts were generated as described previously (62, 63). Briefly, freshly dissected lower limbs recovered from euthanized mice were washed using α-modified Eagle’s medium (α-MEM, Gibco, 32,571–036) to acquire bone marrow cells. Following red blood cell lysis, bone marrow cells were supplemented with α-MEM containing 1% penicillin/streptomycin (Sigma), 10% fetal bovine serum (FBS, Hyclone), and M-CSF (30 ng/ml, R&D Systems, 416-ML-050). After 24 h, nonadherent cells were collected and cultured in medium containing M-CSF for 5 days. Adherent cells were considered BMMs, and seeded on 96-well-plates, and cultured with M-CSF (30 ng/ml) and RANKL (10 ng/ml, R&D Systems, 462-TEC-010) for 5 days. For TRAP staining, cells were fixed with 4% paraformaldehyde for 15 min and stained using a TRAP kit (Sigma-Aldrich, 387A-1 KT) according to the manufacturer’s instructions. TRAP-positive cells containing three or more nuclei were considered osteoclasts. To observe F-actin rings, cells were permeabilized with 0.1% Triton X-100 for 15 min, washed with PBS, covered with 2.5% BSA for 40 min at 37 °C, and then incubated with FITC-labeled phalloidin (Sigma) for 40 min at 37 °C with nuclei stained with DAPI (Beyotime). Images of F-actin rings were obtained using an inverted fluorescence microscope (Leica, DMLS).

### Cellular immunofluorescence

Cellular immunofluorescence was performed as described previously (64). Briefly, BMMs were seeded onto glass coverslips and cultured with M-CSF and RANKL for varying periods of time as described in the text. Cells were fixed with 4% paraformaldehyde for 15 min at room temperature. After permeabilization with 0.1% Triton X-100 for 10 min, samples were blocked with 2.5% BSA at 37 °C for 1 h and then incubated with primary anti-IRF5 rabbit IgG (Abcam, ab181553, 1:100) at 4 °C. After overnight incubation, cells were washed with PBS, incubated with Dylight 594-conjugated goat anti-rabbit immunoglobulin G (Abbkine, A23420) at 37 °C for 1 h, and then stained with FITC-labeled phalloidin (Sigma) at 37 °C for 40 min. After washing with PBS, the glass coverslips were coated with mounting medium containing DAPI (Beyotime), and cells imaged using a confocal laser scanning microscope (Olympus).

### Bone resorbing activity *in vitro*

To analyze the bone resorbing activity of osteoclasts *in vitro*, BMMs were seeded on Corning Osteo Assay Surface multiple well plates or bovine cortical bone slices (Immunodiagnostic Systems) and cultured with M-CSF and RANKL for 8 and 6 days, respectively. Cells on Corning Osteo Assay Surface multiple well plates were removed with 10% NaClO for 5 min at room temperature, air-dried the plates, and imaged the resorption pits with an inverted microscope (63). Alternatively, resorption pit area was quantified by first staining osteoclasts with FITC-labeled phalloidin and DAPI, and imaging F-actin rings by confocal laser scanning microscopy (Olympus). Then, osteoclasts were removed from the bone slices with a cotton swab, and bone samples sonicated in PBS to remove residual cell debris. Bone slices were then stained with 20ug/ml WGA-lectin (Sigma-Aldrich, L-3892) for 45 min and incubated with 3,3′-diaminobenzidine (for 5 min at room temperature (65). Pit areas were quantitated using ImageJ software (version 1.51(100); https://imagej.nih.gov/ij/).

### Preparation of conditioned media from osteoclast-lineage cells

Conditioned media was prepared from BMMs, preosteoclasts or osteoclasts, respectively. BMMs were harvested as described before, with preosteoclasts generated after 1 day culture with 30 ng/ml M-CSF and 10 ng/ml RANKL, and osteoclasts generated after a further 4 to 5 days culture period (48). Cells were then cultured in serum-free medium containing the same concentrations of M-CSF and RANKL for 1 day. The conditioned medium was then collected, centrifuged at 2500 rpm for 10 min at 4 °C, and stored at −80 °C (17).

### *In vitro* migration assays

For wound-healing assays, the mouse MC3T3-E1 osteoblast cell line (ATCC catalog CRL-2594) was used. MC-3T3E1 cells were cultured to confluence in 6-well plates and starved in serum-free α-MEM for 24 h. An artificial wound was created using a pipette tip; the cultures were washed with PBS to remove debris and subsequently treated with serum-free conditioned media prepared from BMM, preosteoclasts or osteoclasts for 24 h. The cells were then fixed in 4% paraformaldehyde and stained with 0.1% crystal violet (Beyotime). Representative fields were captured digitally, and quantified by counting five random fields per well using a light microscope at × 200 magnification (66).

For Transwell migration assays, the two-chamber Transwell-24 well plates (Corning Inc.) with 8 μm pore filters were used. Briefly, serum-free conditioned medium from BMMs, preosteoclasts or osteoclasts were added to the lower compartments of the Transwells, and MC-3T3E1 cells suspended at a final concentration of 1 × 10^5^ cells/ml in serum-free α-MEM seeded in the upper chambers. After a 24 h culture period at 37 °C in 5% CO_2_/95% air, the cells were fixed in 10% formaldehyde for 15 min at room temperature, the non-migrating cells from the upper surface were removed with cotton swabs, and the cells that migrated through the pores to the lower surface were stained with 0.1% crystal violet for 15 min. The migrated cells were quantified by counting five random fields per well using a light microscope (Leica, DMLS) at ×200 magnification (17, 67).

### *In vitro* BMSC culture

BMSCs were cultured as previously reported (68). Briefly, femurs and tibias from 4-6-week-old male mice were separated, the marrow cells flushed with a-MEM and filtered through a 70 μm nylon strainer. Then, the cell suspension was seeded in 10 cm tissue culture dishes and cultured with a-MEM supplemented with 1% penicillin/streptomycin and 10% FBS in a humidified atmosphere of 5% CO_2_/95% air at 37 °C. The medium was changed every 2 to 3 days to remove non-adherent cells, and after 5 days, cells reached approximately 80% confluence. Passage one cells were used for all experiments.

### CFU-F assay

BMSCs were seeded at 2 × 10^4^ cells per well in a 24-well plate. After 21 days culture in BMSC medium, cells were fixed in 4% paraformaldehyde and stained with 0.1% crystal violet (Beyotime) and then photographed. Cell-bound crystal violet was then eluted with 95% ethanol, and optical density measured at 590 nm (69).

### CFU-OB assay

BMSCs were seeded at 2 × 10^4^ cells per well in a 24-well plate with osteogenic medium [*i.e.*, α-MEM containing 1% penicillin/streptomycin, 10% FBS with 10 mM β-glycerophosphate (Sigma-Aldrich) and 50 μg/ml L-ascorbic acid (Sigma-Aldrich)], with media changes every 3 days. After a 21-days culture period, cells were fixed in 4% paraformaldehyde and stained with 40 mM Alizarin red S (Sigma-Aldrich) for 10 min at room temperature and photographed under an inverted microscope (Leica, DMLSCultures were then treated with 10% w/v cetylpyridinium chloride (Sigma-Aldrich) to dissolve the bound Alizarin red S, and optical density measured at 562 nm (70).

### Assessment of osteoblast mineralization

For indirect co-culture, MC-3T3E1 osteoblast-like cells were seeded at a density of 2 × 10^4^ cells per well in 24-well plates, and cultured with serum-containing conditioned medium from BMMs, preosteoclasts or osteoclasts in the presence of osteogenic medium in a 1:1 ratio with media changed every 3 days for up to 21 days total (70).

For direct co-culture, BMSCs were seeded at 2 × 10^4^ cells per well in a 24-well plate for co-culture. BMM-derived osteoclasts were added to the adherent osteoblasts (1 × 10^5^ cells per well) in BMSC medium. After 24 h, osteogenic medium was added with medium changes every 3 days for up to 21 days in culture (71, 72). The assay for mineralized nodules was performed as described above.

### RNA-seq analysis

RNA was isolated from whole cell lysates with TRIzol reagent (Ambion, 15,596,026), RNA quality was determined by Bioanalyzer (Agilent), libraries were sequenced on a BGISEQ-500RS (73). Sequencing reads were mapped to the mouse reference genome (mm10/GRCm38) using STAR v.2.7.2 b (74). Gene expression counts were calculated using HTSeq v.0.9.1 (75). DESeq2 was used to estimate significance between any two experimental groups (76). Gene Ontology analysis was analyzed using the DAVID Bioinformatics Resources 6.8 (77). The data has been uploaded to the Gene Expression Omnibus database with the login number GSE297530.

### Quantitative reverse transcription–PCR analysis

Total RNA was isolated from cultured cells with TRIzol reagent (Ambion, 15,596,026). Mouse complementary DNA was reverse-transcribed from 2.0 μg total RNA with EvoScript Universal complementary DNA Master (Roche, 07,912,439,001). Quantitative reverse transcription–PCR was did by ChamQ™ SYBR Quantitative reverse transcription–PCR Master Mix kit (Vazyme, Q311–02) using the Bio-Rad CFX96™ machine. Primer sequences are as followings:

*Irf5* (5′-AATACCCCACCACCTTTTGA-3′, 5′-TTGAGATCCGGGTTTGAGAT-3′), *Irf8* (5′-ATGGTCATCAGCTTCTACTACG-3′, 5′-ATCCGGCCCATACAACTTAG-3′), *Nfatc1* (5′-CAAGTCTCACCACAGGGCTCACTA-3′, 5′-TCAGCCGTCCCAATGAACAG-3′), *c-Fos* (5′-ACGTGGAGCTGAAGGCAGAAC-3′, 5′-AGCCACTGGGCCTAGATGATG-3′), *Ctsk* (5′-CACCCAGTGGGAGCTATGGAA-3′, 5′-GCCTCCAGGTTATGGGCAGA-3′), *Mmp9* (5′-GCCCTGGAACTCACACGACA-3′, 5′-TTGGAAACTCACACGCCAGAAG-3′), *Mmp14* (5′-TATGGTTTACAAGTGACAGGCA-3′, 5′-AAACTTATCCGGAACACCACAG-3′), *c-Src* (5′-CTATGTGGAGCGGATGAACTAT-3′, 5′-ATTCGTTGTCTTCTATGAGCCG-3′), *Itgb3* (5′-CCCCGATGTAACCTGAAGGAG-3′, 5′-GAAGGGCAATCCTCTGAGGG-3′), *Pdgfb* (5′-GTCCAGGTGAGAAAGATTGAGA-3′, 5′-GTCATGGGTGTGCTTAAACTTT-3′), *Tnfsf11* (5′-TGTACTTTCGAGCGCAGATG-3′, 5′-AGGCTTGTTTCATCCTCCTG-3′), *Tnfrsf11b* (5′-CTTGCCTTGATGGAGAGCCT-3′, 5′-TCGCTCGATTTGCAGGTCT-3′), *Il1b* (5′-ATCTTTTGGGGTCCGTCAACT-3′, 5′-GCAACTGTTCCTGAACTCAACT-3′), *Il6* (5′-TGTATGAACAACGATGATGCACTT-3′, 5′-ACTCTGGCTTTGTCTTTCTTGTTATCT-3′), *Il12b* (5′-TGGTTTGCCATCGTTTTGCT-3′, 5′-TCACTGTTTCTCCAGGGGCA-3′), *Tnf* (5′-CACGCTCTTCTGTCTACTGA-3′, 5′-ATCTGAGTGTGAGGGTCTGG-3′).

### Western blot

Western blot was performed as described previously (65). Briefly, cells were washed with cold PBS and lysed with a RIPA lysate buffer containing protease and phosphatase inhibitors (Beyotime). Protein concentration was determined with a BCA test kit (Applygen, P1511–1) according to the manufacturer’s instruction. Total proteins were transferred onto a PVDF membrane and incubated overnight with specific primary antibodies at 4 °C. GAPDH served as the loading control. Western blot was performed using the following antibodies: anti-IRF5 rabbit IgG (Abcam, ab181553, 1:1000), anti-IRF8 rabbit IgG (ABclonal, A5798, 1:500), anti-Ctsk mouse IgG (Santa Cruz, sc-48353, 1:500), anti-c-Fos mouse IgG (Santa Cruz, sc-166940, 1:500), anti-NFATc1 mouse IgG (Santa Cruz, sc-7294, 1:500), anti-C-SRC rabbit IgG (Cell Signaling Technology, 2109, 1:1000), anti-Mmp9 rabbit IgG (Abcam, ab38898, 1:1000), anti-Mmp14 rabbit IgG (Abcam, ab51074, 1:5000), anti-Integrin-β3 rabbit IgG (Cell Signaling Technology, 4702, 1:1000).

### Statistical analysis

All values were expressed as mean ± SD. Unpaired Student’s *t* test was used to analyze the statistical difference between two groups, one or two-way ANOVA with Bonferroni correction was used to determine differences among multiple groups. A *p* value of less than 0.05 was considered statistically significant.

## Data availability

All data for this study are contained within the manuscript and its supporting information.

## Supporting information

This article contains supporting information.

## Conflict of interest

The authors declare that they have no conflicts of interest with the contents of this article.
