# Supplementary material for: Myeloid-specific interferon regulatory factor 5 promotes bone formation via orchestration of osteoclast lineage-osteoblast coupling
Source: J Biol Chem. 2026 Jun 22;302(8):113279. doi: 10.1016/j.jbc.2026.113279 (PMC13400275; doi:10.1016/j.jbc.2026.113279)
Supplement: Supporting Information [file mmc1.docx]

**Supplementary Materials for**

**Myeloid-specific interferon regulatory factor 5 promotes bone formation via orchestration of osteoclast lineage-osteoblast coupling**

Huan Zhao^1^, Xiaoyue Sun^1^, Songqin Zhou^1^, Zhengrui Chang^1^, Jingwen Yang^1^, Jingjing Yu^1^, Zijun Wang^1^, Yi Tang^2,3^, Changdong Lin^4^, Li Wang^1^*, Stephen J. Weiss^2,3^, Lingxin Zhu^1^*

*Corresponding Authors:

E-mail: dentist-wang@whu.edu.cn (L.W.); lingxin.zhu@whu.edu.cn (L.Z.)

**The PDF file includes:**

Fig. S1. IRF5 expression during osteoclastogenesis.

Fig. S2. IRF5 expression in osteoblasts.

Fig. S3. Impact of myeloid-specific *Irf5* targeting on bone mass in female mice.

Fig. S4. Impact of myeloid-specific *Irf5* targeting on osteoclast differentiation *in vitro*.

Fig. S5. Impact of *Irf5* global knockout on osteoclast differentiation *in vitro*.

Fig. S6. Myeloid *Irf5* deficiency does not affect RANKL or OPG secretion by osteoblasts.

Fig. S7. Impact of myeloid *Irf5* on osteoblast progenitor abundance.

Fig. S8. Impact of myeloid *Irf5* deletion on inflammatory response.

Fig. S9. Genome wide analysis of transcriptional programs in *Irf5^-/-^* osteoclasts.

The raw images for Western blots shown in Fig.1E, Fig.2B, Fig.3A, Fig.3B and Fig.S2B.

**
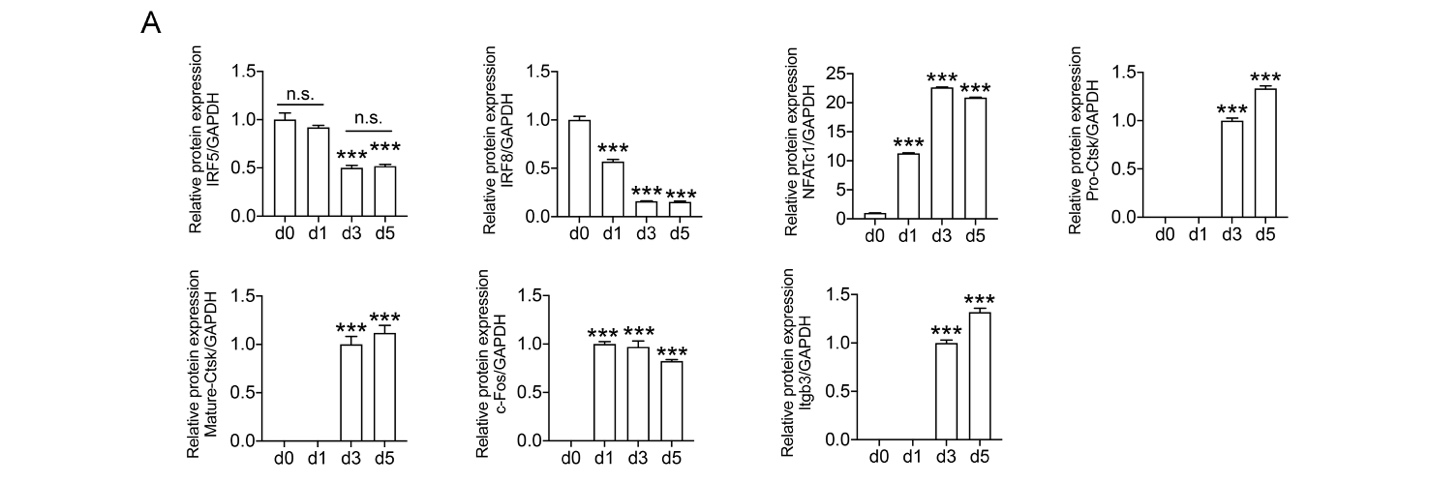
**

**Fig. S1. IRF5 expression** **during osteoclastogenesis.** (A) Relative protein expression levels of IRF5, IRF8, NFATc1, c-Fos, Ctsk and Itgb3 ﻿in BMMs at 0, 1, 3, and 5 days after M-CSF and RANKL stimulation as assessed in Fig 1E by ﻿Image J software. *** *P*<0.001; n.s., no significance. Data analyzed using one-way ANOVA with Bonferroni correction are presented as mean ± SD (n = 3).


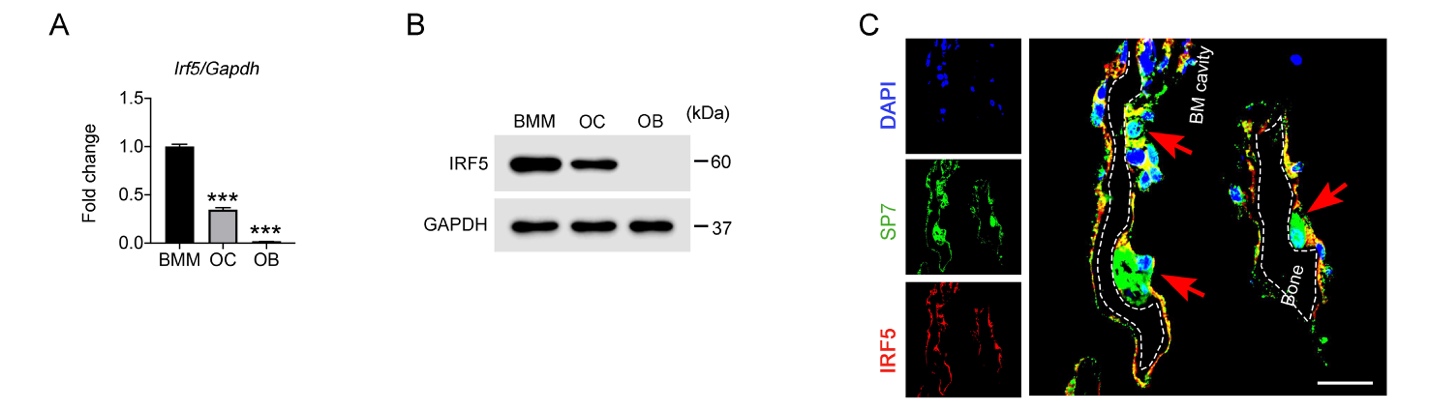


**Fig. S2. IRF5 expression in osteoblasts.** (A) Relative mRNA expression of *Irf5* in BMMs, OCs and OBs. *** *P*<0.001. Data are presented as mean ± SD (n = 3). ﻿Data analyzed using one-way ANOVA with Bonferroni correction. (B) Western blot of IRF5 in BMMs, OCs and OBs (n = 3). (C) IRF5 (red) and SP7 (green) as assessed by immunofluorescent staining of femur frozen sections from 3–4-week-old wild-type male mice (n = 3). Red solid arrow indicates osteoblasts on the bone surface. Scale bar, 20μm.


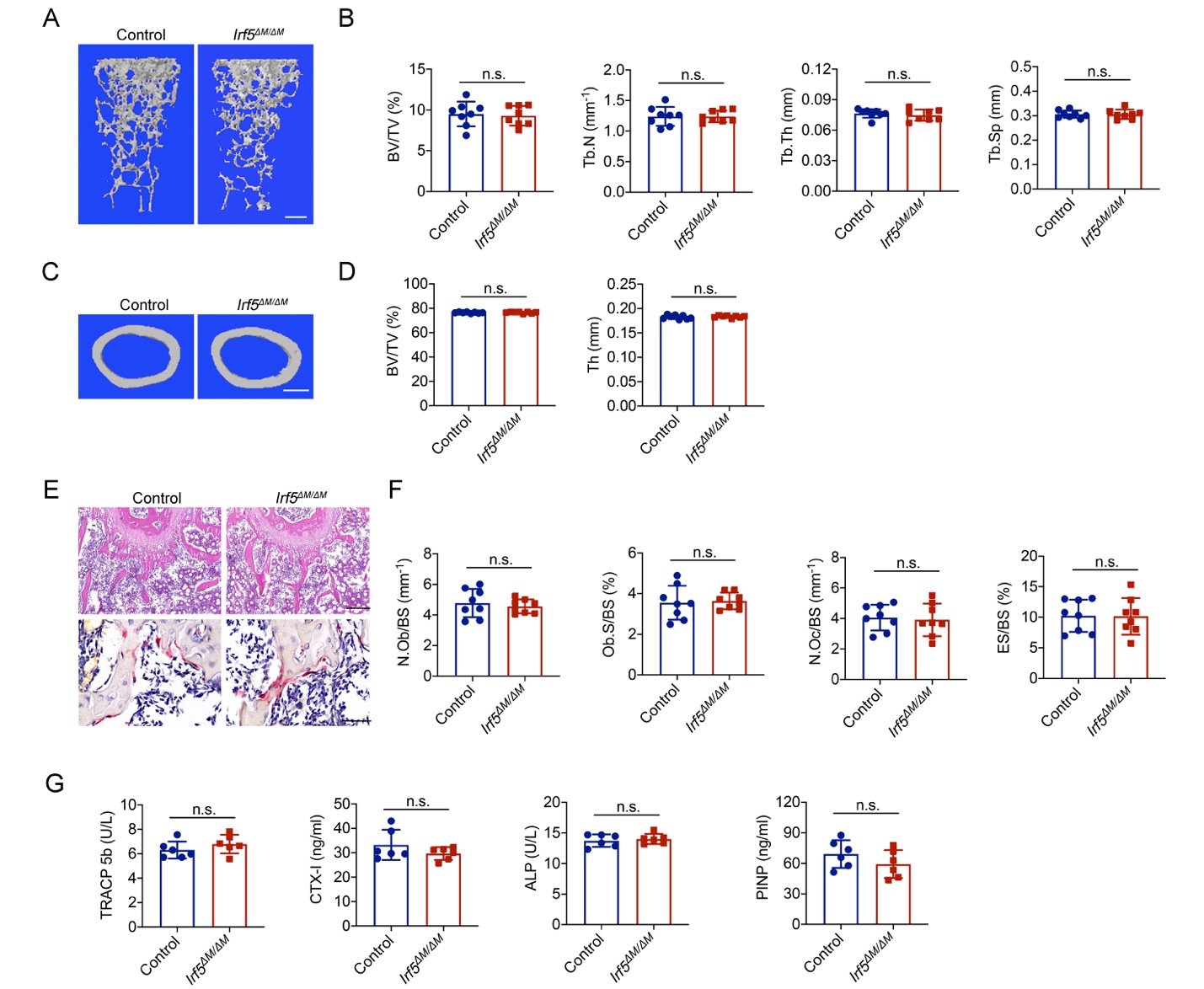


**Fig. S3. Impact of myeloid-specific *Irf5* targeting on bone mass in female mice.** (A) Representative 3D reconstruction images of ﻿distal femur trabeculae of 3-month-old control and *Irf5^ΔM/ΔM^* female mice are shown. Scale bars, 500μm, (n = 8). (B) Quantification of BV/TV, Tb.N, Tb.Th, Tb.Sp of trabeculae bone as determined by μCT in 3-month-old female control and *Irf5^ΔM/ΔM^* mice (n = 8). (C) Representative 3D reconstructions of ﻿distal femur cortical bone of 3-month-old control and *Irf5^ΔM/ΔM^* female mice are shown. Scale bars, 500μm, (n = 8). (D) Quantification of BV/TV, Thickness (Th) of cortical bone as determined by μCT in 3-month-old female control and *Irf5^ΔM/ΔM^* mice (n = 8). (E) H&E (up) and TRAP (down) staining of distal femur trabeculae of 3-month-old female control and *Irf5^ΔM/ΔM^* mice. Scale bar, 200μm (H&E), 40μm (TRAP), (n = 8). (F) Quantification of N.Ob/BS, Ob.S/BS, N.Oc/BS and ES/BS as determined by H&E and TRAP staining (n = 8). (G) Serum TRACP 5b, CTX-I, ALP and PINP levels in 3-month-old female control and *Irf5^ΔM/ΔM^* mice (n = 6). n.s., no significance. Data analyzed using unpaired Student’s *t* test are presented as mean ± SD.

**
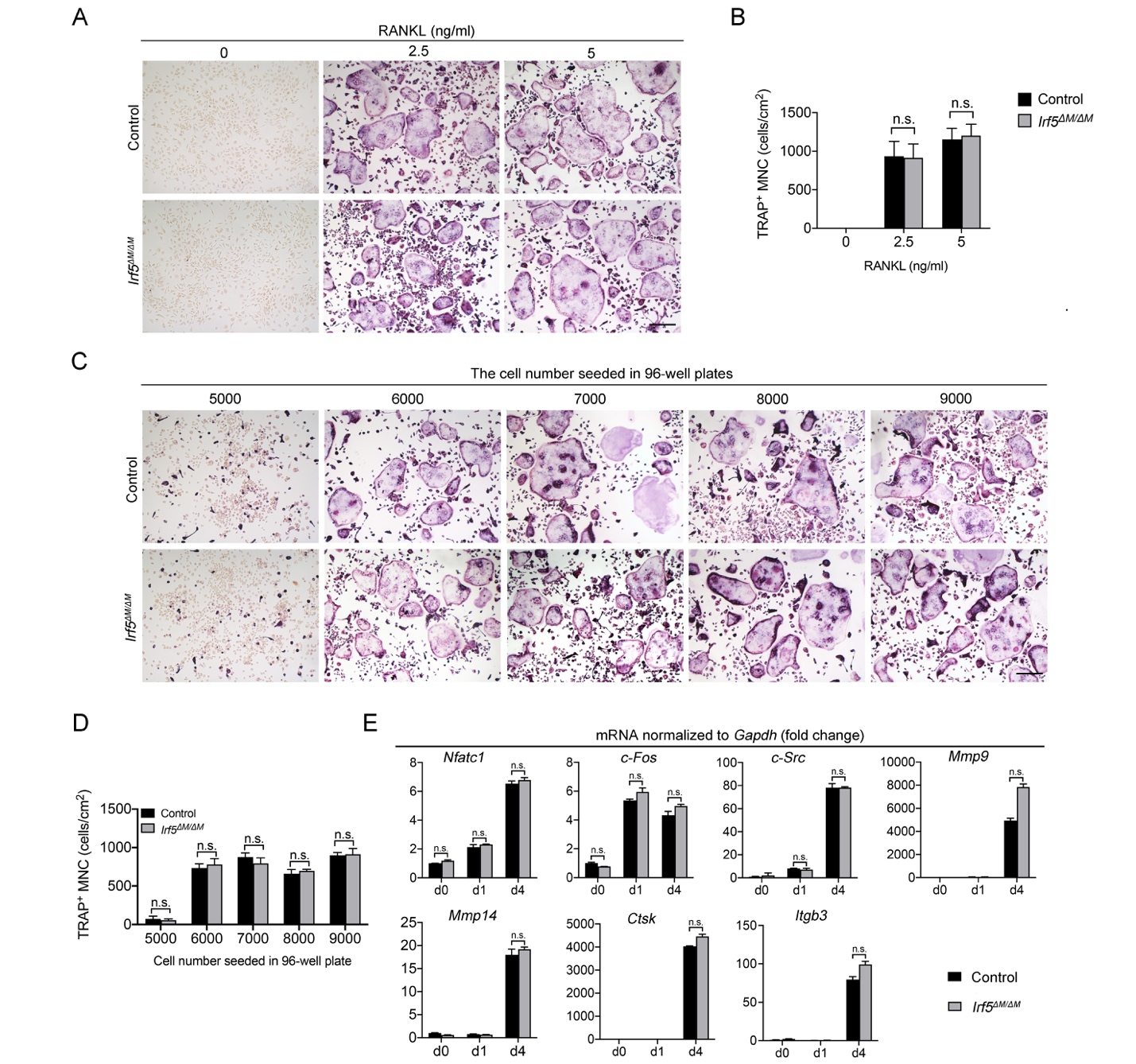
**

**Fig. S4. Impact of** **myeloid-specific *Irf5* targeting on osteoclast differentiation *in vitro*.** (A) TRAP staining of control and *Irf5^ΔM/ΔM^* cells in response to varying concentrations of RANKL as indicated, scale bars, 200μm, (n = 3). (B) TRAP^+^ MNCs in (A) were quantitated (n = 3). (C) TRAP staining of control and *Irf5^ΔM/ΔM^* cells as a function of initial cell densities plated as indicated, scale bars, 200μm (n = 3). (D) ﻿﻿TRAP^+^ MNCs in (C) were ﻿quantitated (n = 3). (E) Relative mRNA expressions of *Nfatc1*, *c-Fos*, *c-Src, Mmp14, Mmp9, Ctsk*, *Itgb3* ﻿in BMMs at 0, 1, and 4 days after M-CSF and RANKL stimulation of control and *Irf5^ΔM/ΔM^* cells (n = 3). Data analyzed using two-way ANOVA with Bonferroni correction are presented as mean ± SD. n.s., no significance.


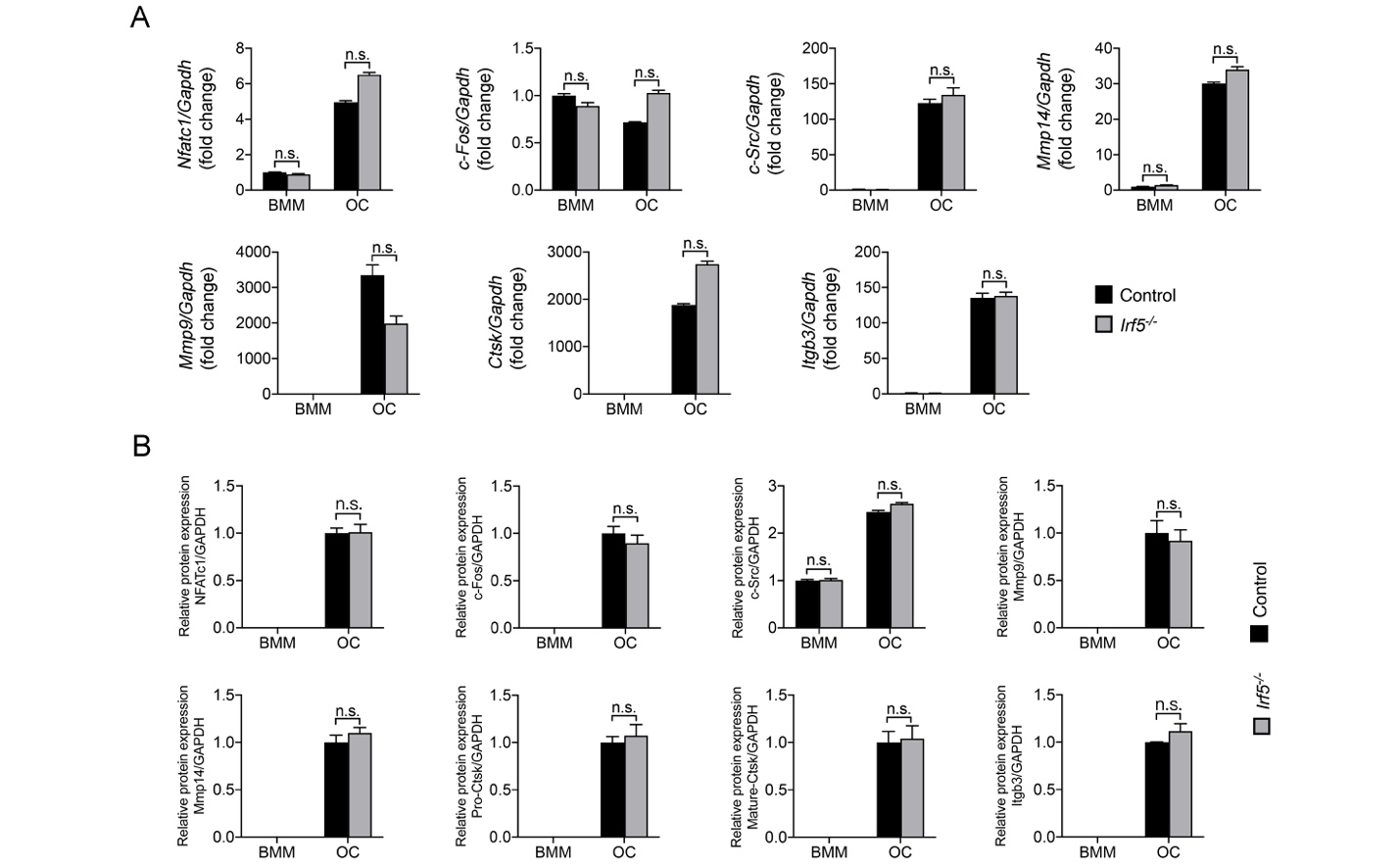


**Fig. S5.**  **Impact of *Irf5* global knockout** **on osteoclast differentiation *in vitro.*** (A) Relative mRNA expressions of *Nfatc1*, *c-Fos*, *c-Src, Mmp14, Mmp9, Ctsk*, *Itgb3* ﻿in BMMs and OCs of control and *Irf5^-/-^* group (n = 3). ﻿(B) The relative protein expression levels IRF5, IRF8, NFATc1, c-Fos, Ctsk and Itgb3 in BMMs and OCs of control and *Irf5^-/-^* group are shown as assessed in Fig 3B by ﻿Image J software (n = 3). Data analyzed using two-way ANOVA with Bonferroni correction are presented as mean ± SD. n.s., no significance.

**
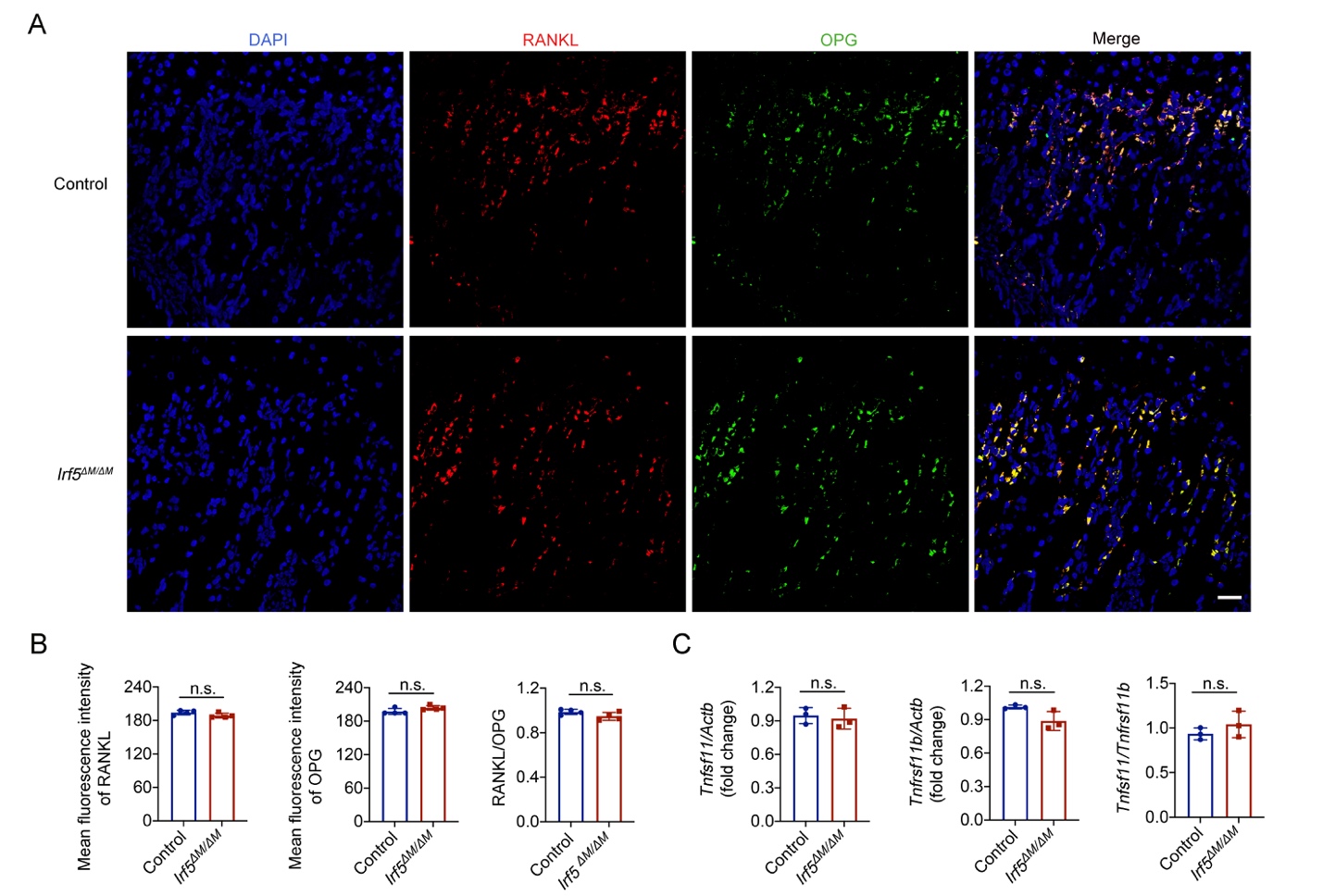
**

**Fig. S6. Myeloid *Irf5* deficiency does not affect RANKL or OPG secretion by osteoblasts.** (A) RANKL (red) and OPG (green) as assessed by immunofluorescent staining of femur sections from 4–6-week-old control and *Irf5^ΔM/ΔM^* male mice, DAPI (blue), scale bars, 20μm, n = 4. (B) The mean fluorescence intensity of RNAKL and OPG was analyzed by ﻿Image J software (n = 4). (C) Relative mRNA expression of *Tnfsf11* and *Tnfrsf11b* in BMSC-derived osteoblasts from long bones of 4–6-week-old male control and *Irf5^ΔM/ΔM^* mice (n = 3). n.s., no significance. Data analyzed using unpaired Student’s *t* test are presented as mean ± SD.

**
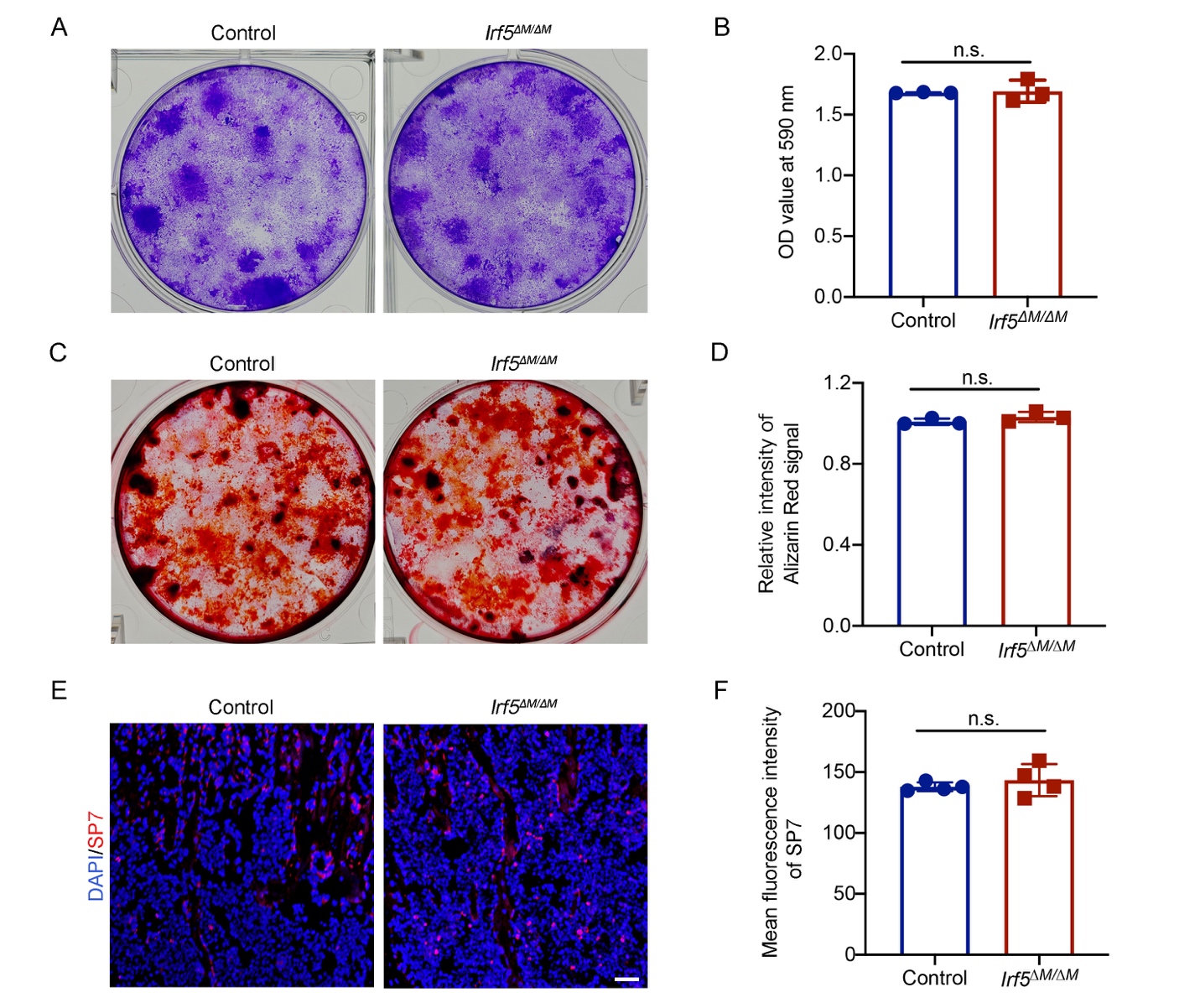
**

**Fig. S7. Impact of myeloid *Irf5* on osteoblast progenitor abundance.** (A)﻿ Colony-forming unit-fibroblasts (CFU-Fs) of BMSCs isolated from long bones of 4–6-week-old male mice. Cells were stained with 0.1% crystal violet on day 21 in culture (n = 3). (B) Quantitative analysis of the eluted crystal violet solution by measuring the optical density at 590 nm (n = 3). (C)﻿ ﻿ Colony-forming unit-osteoblasts (CFU-OBs) of BMSCs isolated from long bones of 4–6-week-old male mice. Cells were stained with Alizarin red on day 21 in culture (n = 3). (D) ﻿The relative intensity of Alizarin red was determined using Image J software (n = 3). (E) SP7 (red) as assessed by immunofluorescent staining of femur sections from 4–6-week-old control and *Irf5^ΔM/ΔM^* male mice, DAPI (blue), scale bars, 20μm, n = 4. (F) The mean fluorescence intensity of SP7 was analyzed by ﻿Image J software (n = 4). n.s., no significance. Data analyzed using unpaired Student’s *t* test are presented as mean ± SD.

**
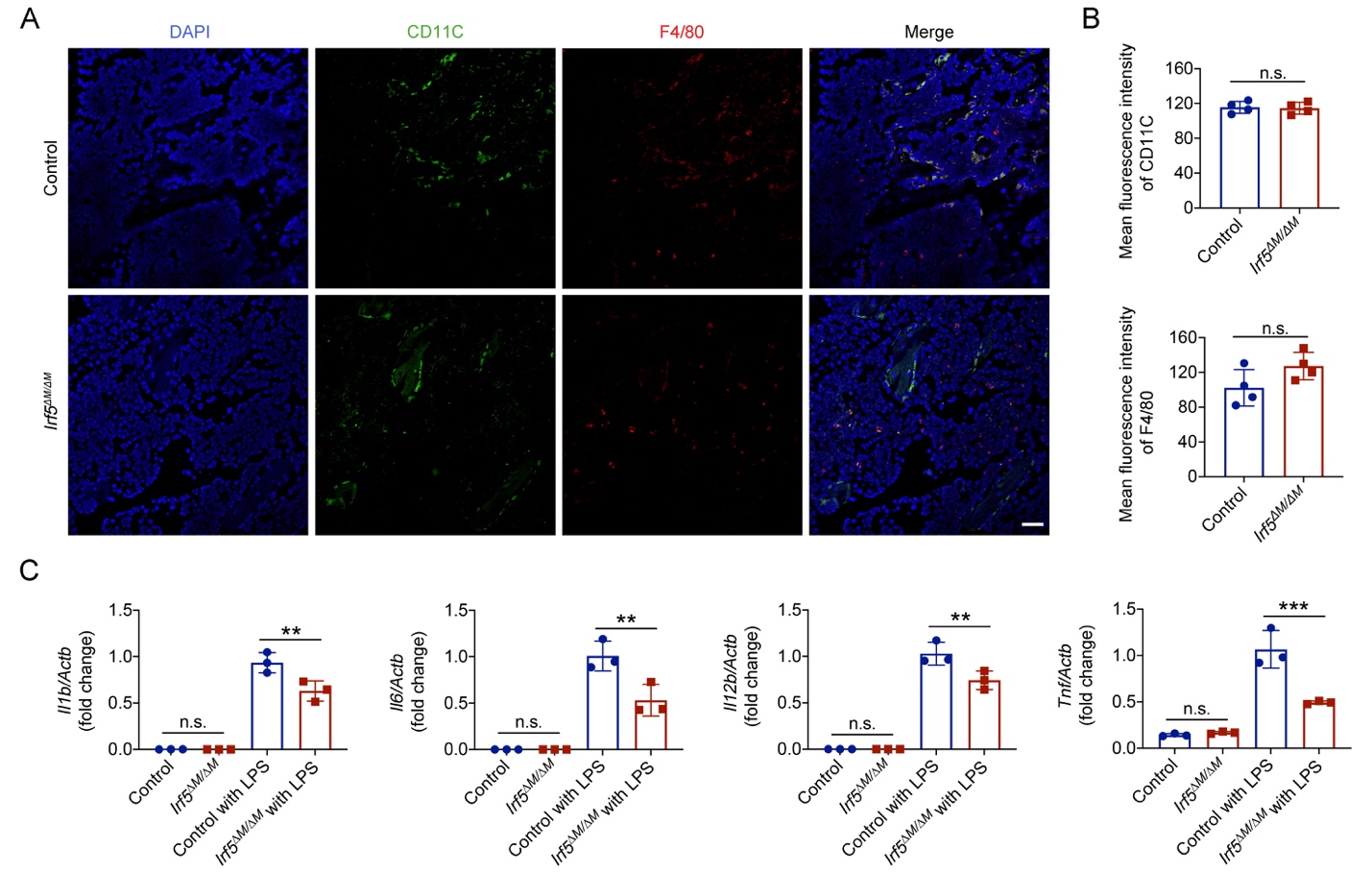
**

**Fig. S8. Impact of myeloid *Irf5* deletion on inflammatory response.** (A) CD11C (green) and F4/80 (red) as assessed by immunofluorescent staining of femur sections from 4–6-week-old control and *Irf5^ΔM/ΔM^* male mice, DAPI (blue), scale bars, 20μm, n = 4. (B) The mean fluorescence intensity of CD11C and F4/80 were analyzed by ﻿Image J software (n = 4). (C) Relative mRNA expression *of ﻿Il1b*, *Il6*, *﻿Il12b* and *Tnf* in BMMs from long bones of 4–6-week-old male control and *Irf5^ΔM/ΔM^* mice with or without stimulation of 100 ng/ml LPS for 12 h (n = 3). n.s., no significance, ** *P*<0.01, *** *P*<0.001. Data analyzed using unpaired Student’s *t* test (B) or one-way ANOVA with Bonferroni correction (C) are presented as mean ± SD.

**
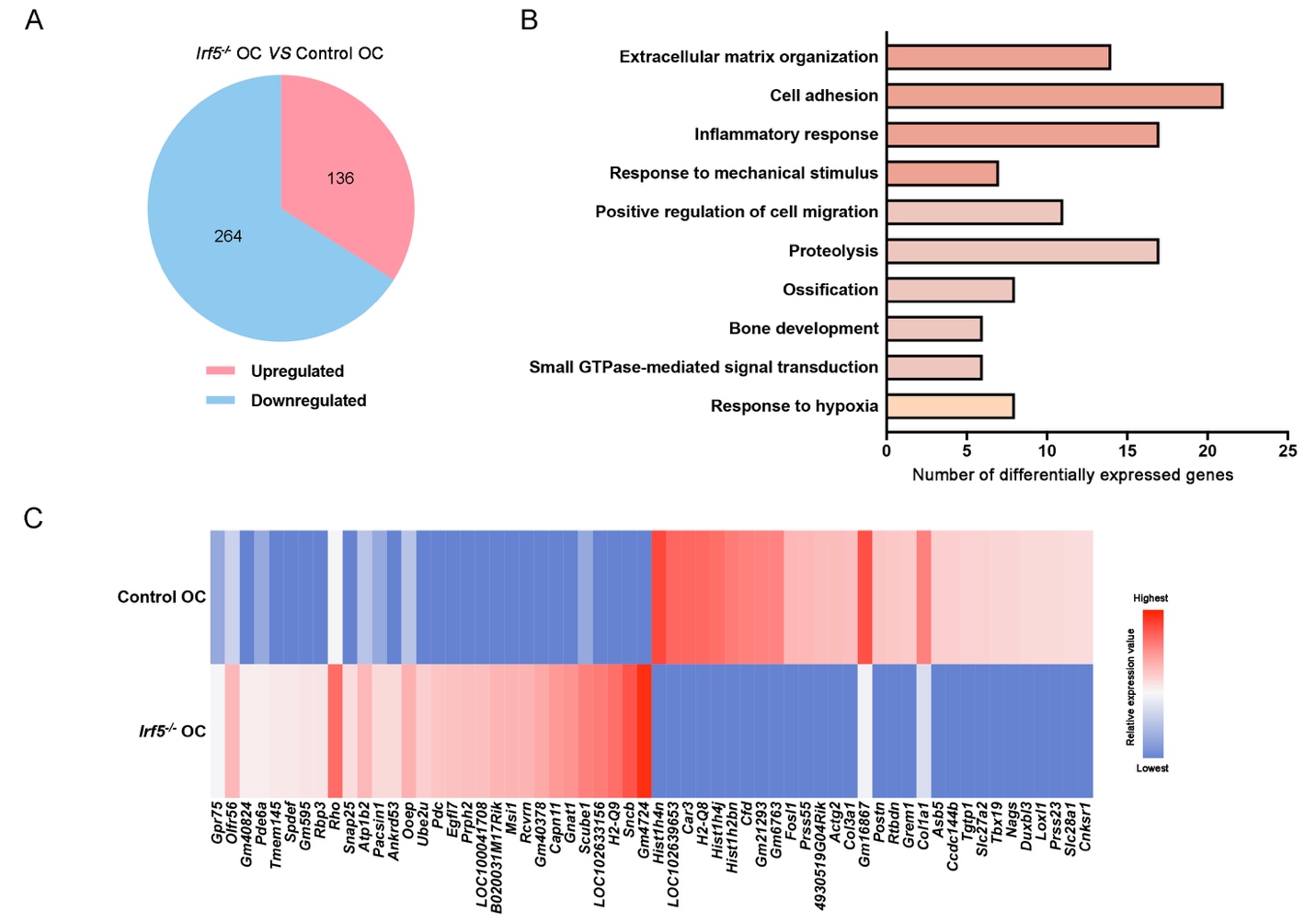
**

**Fig. S9. Genome wide analysis of transcriptional programs in *Irf5^-/-^* osteoclasts.** (A) ﻿Pie chart distribution of total transcripts altered in *Irf5^-/-^* OC relative to control OC cells. (B) ﻿DAVID GO analysis of differentially expressed genes from *Irf5^-/-^* OC versus control OC cells. (C) ﻿The 30 most highly upregulated and downregulated transcripts in *Irf5^-/-^* OC as compared with control OC cells are shown with fold-changes. ﻿


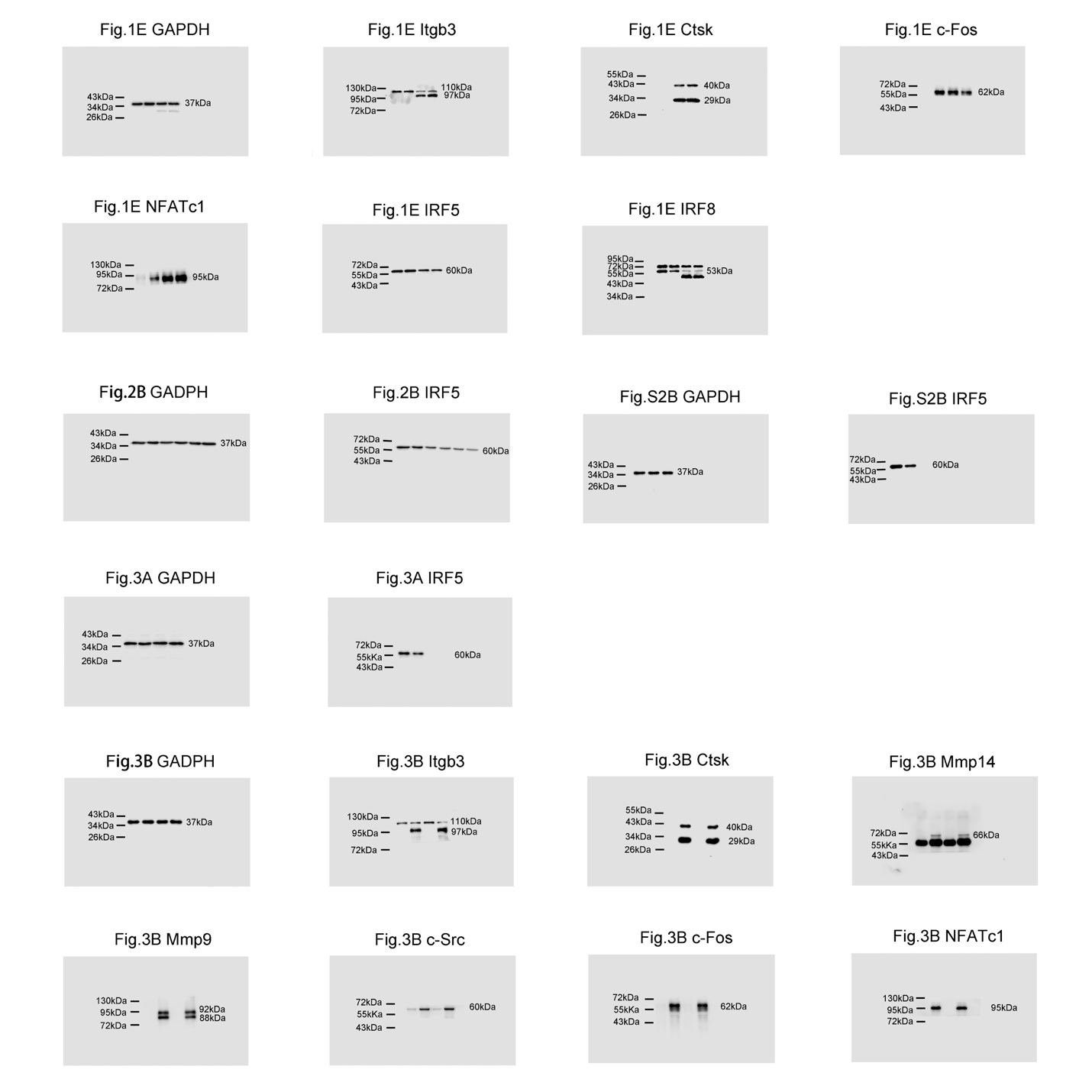


The raw images for Western blots shown in Fig.1E, Fig.2B, Fig.3A, Fig.3B and Fig.S2B.
